# Supplementary material for: The preprophase band-associated kinesin-14 OsKCH2 is a processive minus-end-directed microtubule motor
Source: Nat Commun. 2018 Mar 14;9:1067. doi: 10.1038/s41467-018-03480-w (PMC5852081; doi:10.1038/s41467-018-03480-w)
Supplement: Supplementary file 1 — Supplementary Information [file 41467_2018_3480_MOESM1_ESM.pdf]

Supplementary Information

Supplementary Figures 1-8

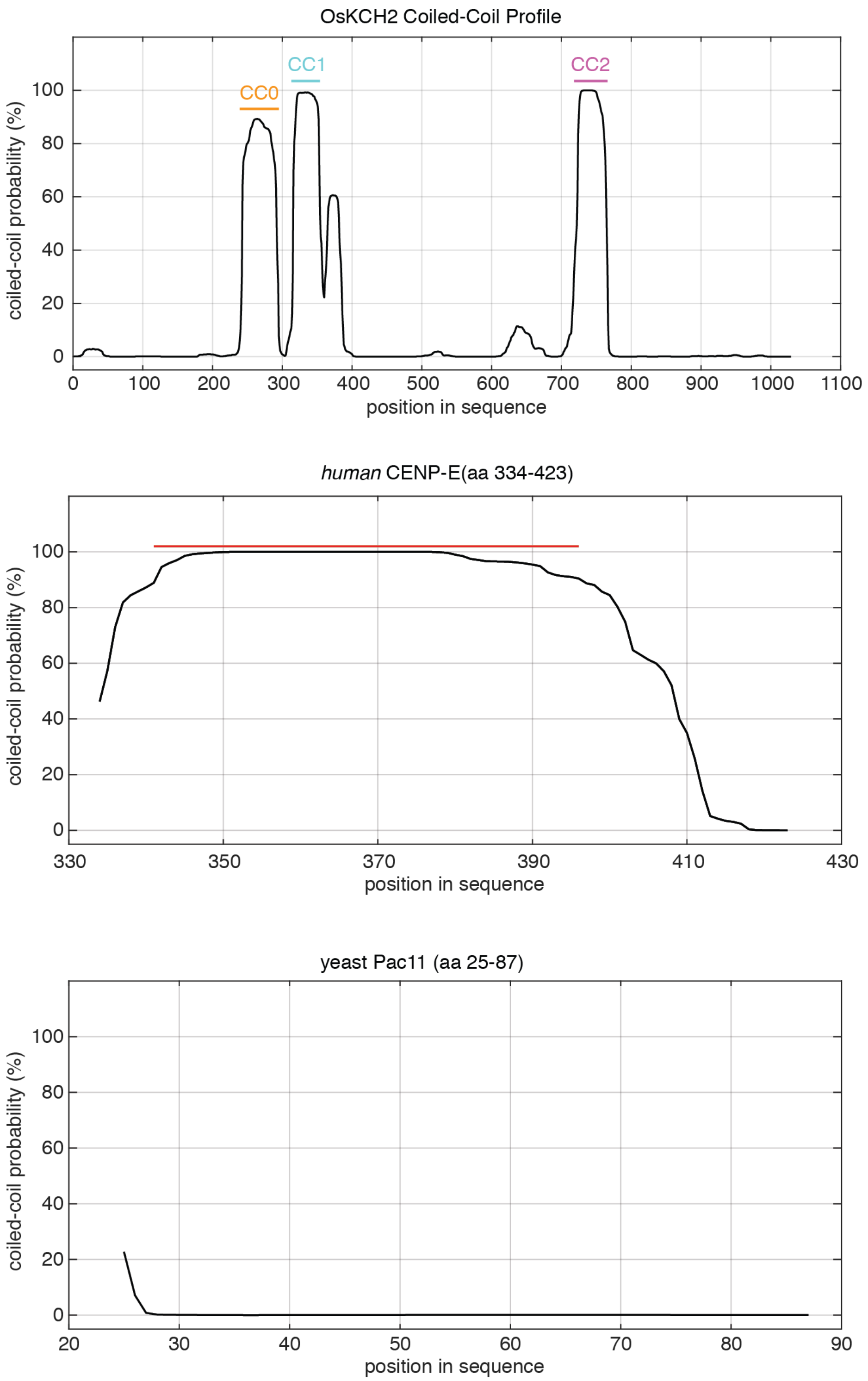

Supplementary Figure 1: Predicted coiled-coil motifs of OsKCH2. (Top) Based on the prediction by the program MARCOIL with a coiled-coil cutoff probability of 80%, the full-length OsKCH2 contains three coiled-coils, which include CC0 in aa 239-295, CC1 in aa 313-354, and CC2 in aa 718-766. (Middle) The predicted coiled-coil profile of human kinesin-7 CENP-E(aa 343-423) by MARCOIL. Red line depicts the region that forms an actual coiled-coil structure based on X-ray crystallography[1]. This serves as a positive control of the coiled-coil prediction of OsKCH2 by MARCOIL. (Bottom) The predicted coiled-coil profile by MARCOIL of yeast Pac11(aa 25-87) that was experimentally found to be intrinsically disordered[2]. This serves as a negative control of the coiled-coil prediction of OsKCH2 by MARCOIL[3].

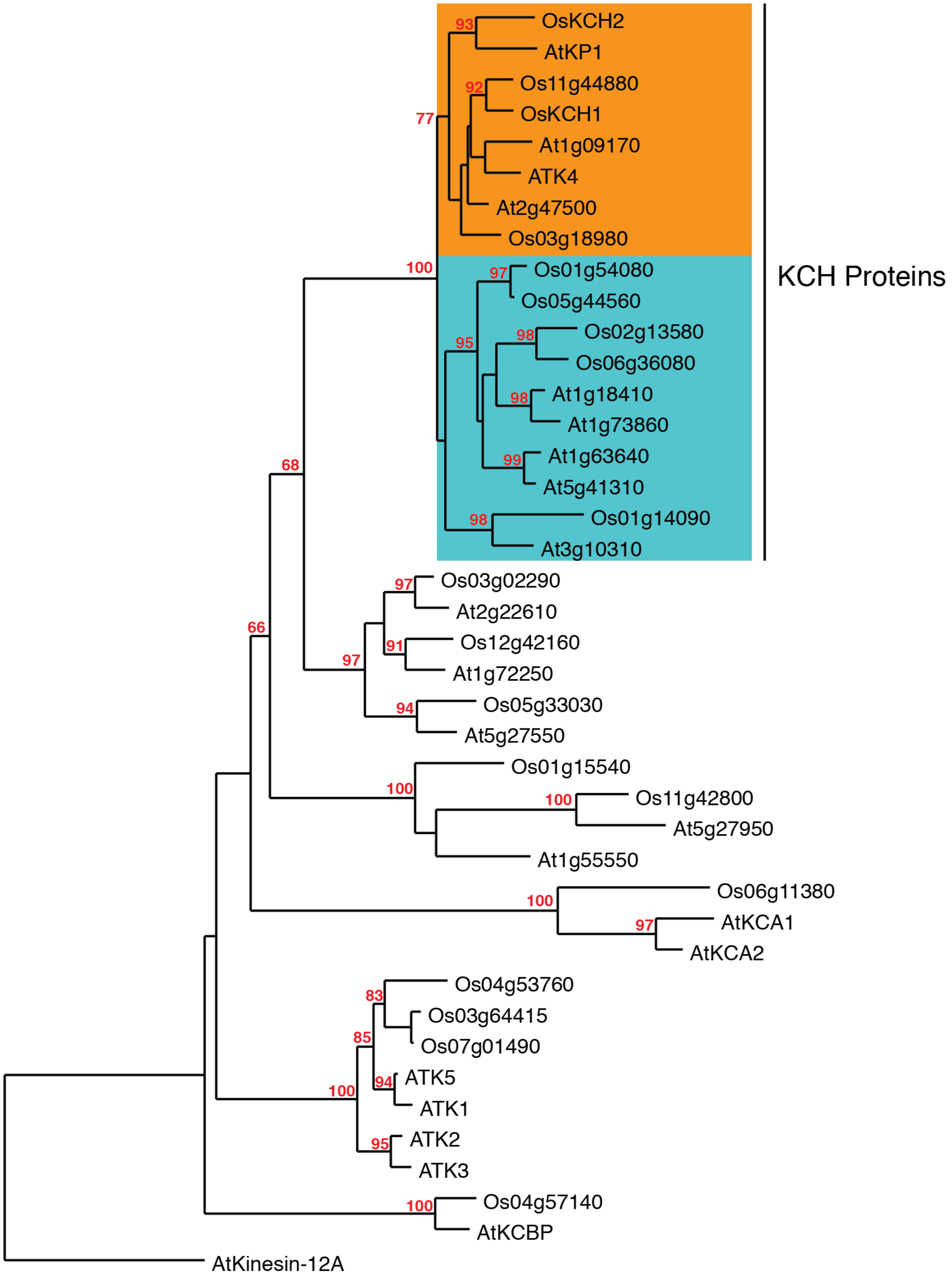

Supplementary Figure 2: Phylogenetic tree of kinesin-14 motors from *Oryza sativa* and *Arabidopsis thaliana*. The phylogenetic tree was generated with the program PhyML (www.phylogeny.fr) based on the full-length protein sequences of the

kinesin-14 family from *Oryza sativa* (japonica cultivar) and *Arabidopsis thaliana*. The maximum likelihood method was used. Branch support values were represented in percentages (%), and only values greater than 60% were shown in the tree. Protein sequence data of *O. sativa* (japonica cultivar) were obtained from MSU RGAP Release 7 (http://rice.plantbiology.msu.edu/; "LOC_" was omitted from each locus call) except for OsKCH2, which was not annotated in the release; Protein sequence data of *A. thaliana* were from TAIR10 (http://www.arabidopsis.org/); AtKCH1=LOC_Os12g36100. AtKP1=At3g44730; ATK1=At4g21270/KATA; ATK2=At4g27180/KATB; ATK3=At5g54670/KATC; ATK4=At5g27000/KATD; ATK5=At4g05190; AtKCA1=At5g10470/KAC1; AtKCA2=At5g65460/KAC2; AtKCBP=At5g65930. AtKinesin-12A/At4g14150 was included as an outgroup protein in the analysis. Complete protein sequence alignment of all kinesin-14s in the phylogenetic tree can be found in Supplementary Data 1.

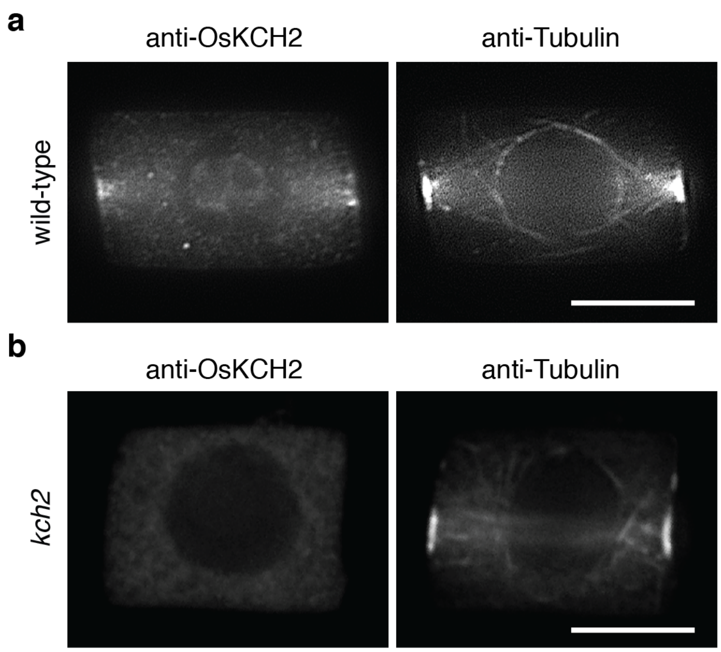

Supplementary Figure 3: OsKCH2 exhibits cortical localization in a rice cell at prophase. a, Immunostaining of a wildtype rice cell using anti-OsKCH2 (left) and anti-Tubulin (right). **b,** Immunostaining of a *kch2* mutant cell using anti-OsKCH2 (left) and anti-Tubulin (right). Scale bar: 5 μm.

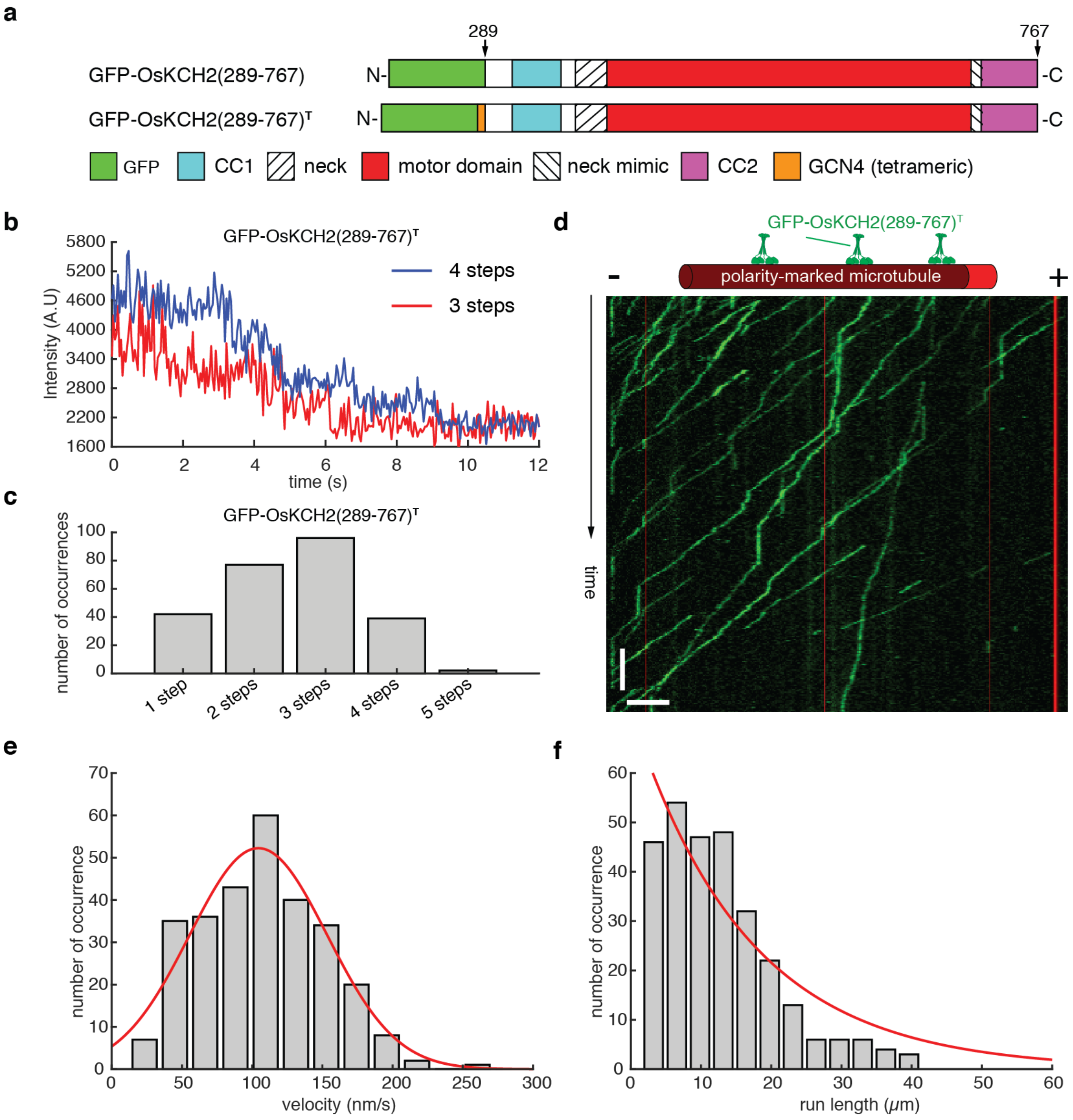

Supplementary Figure 4: The artificial homotetramer GFP-OsKCH2(289-767)^T exhibits ultraprocessive minus-end-directed motility on single microtubules. a, Schematic diagrams of GFP-OsKCH2(289-767) and GFP-OsKCH2(289-767)^T. **b,** Example fluorescence intensity traces over time of individual GFP-OsKCH2(289-767)^T molecules immobilized on the microtubules. **c,** Histogram of the photobleaching steps of GFP-OsKCH2(289-767)^T (n = 255). **d,** Example kymograph showing that individual GFP-OsKCH2(289-767)^T molecules move processively toward the minus-end on a

single microtubule. **e**, Velocity histogram of single GFP-OsKCH2(289-767)^{T} molecules. Red line indicates a Gaussian fit to the velocity histogram. **f**, Run-length histogram of single GFP-OsKCH2(289-767)^{T} molecules. Red line indicates a single-exponential fit to the run-length histogram. Scale bars: 1 minute (vertical) and 5 μm (horizontal).

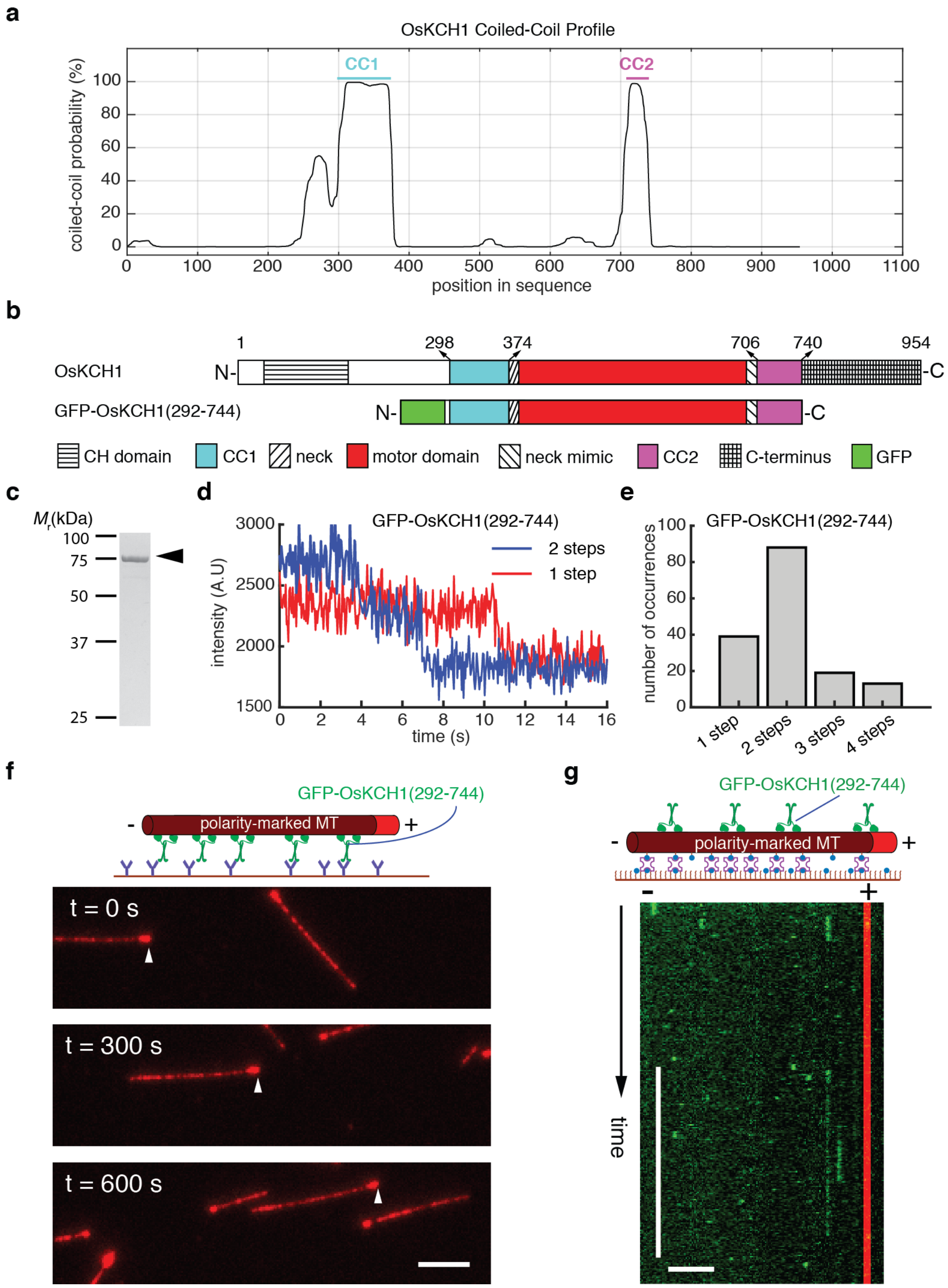
a
OsKCH1 Coiled-Coil Profile
CC1
CC2
coiled-coil probability (%)
100
80
60
40
20
0
0
100
200
300
400
500
600
700
800
900
1000
1100
position in sequence
b
1
298
374
706
740
954
OsKCH1
N-
-C
GFP-OsKCH1(292-744)
N-
-C
CH domain
CC1
neck
motor domain
neck mimic
CC2
C-terminus
GFP
c
M_r(kDa)
100
75
50
37
25
d
GFP-OsKCH1(292-744)
2 steps
1 step
intensity (A.U)
3000
2500
2000
1500
0
2
4
6
8
10
12
14
16
time (s)
e
GFP-OsKCH1(292-744)
number of occurrences
100
80
60
40
20
0
1 step
2 steps
3 steps
4 steps
f
GFP-OsKCH1(292-744)
-
polarity-marked MT
+
t = 0 s
t = 300 s
t = 600 s
g
GFP-OsKCH1(292-744)
-
polarity-marked MT
+
-
+
time

Supplementary Figure 5: GFP-OsKCH1(292-744) is a nonprocessive minus-end-directed microtubule motor. a, Predicted coiled-coil motifs of OsKCH1. Based on the prediction by the program MARCOIL with a coiled-coil cutoff probability of 80%. The full-length OsKCH1 contains an upstream putative coiled-coil (CC1, aa 298-374) and a downstream putative coiled-coil (CC2, aa 706-740). **b,** Schematic diagrams of the full-length OsKCH1 and GFP-OsKCH1(292-744). **c,** Coomassie-stained SDS-PAGE of purified recombinant GFP-OsKCH1(292-744). **d,** Example fluorescence intensity traces over time of individual GFP-OsKCH1(292-744) molecules immobilized on the microtubules. **e,** Histogram of the photobleaching steps of GFP-OsKCH1(292-744) (n = 156). **f,** Micrograph montage showing surface-immobilized GFP-OsKCH1(292-744) molecules drive microtubules to glide with minus-end-directed motility. White arrowheads indicate the plus ends of polarity-marked microtubules. **g,** Example kymograph showing that individual GFP-OsKCH1(292-744) molecules are unable to produce processive motility on a single microtubule. Scale bars: 30 s (vertical) and 5 μm (horizontal).

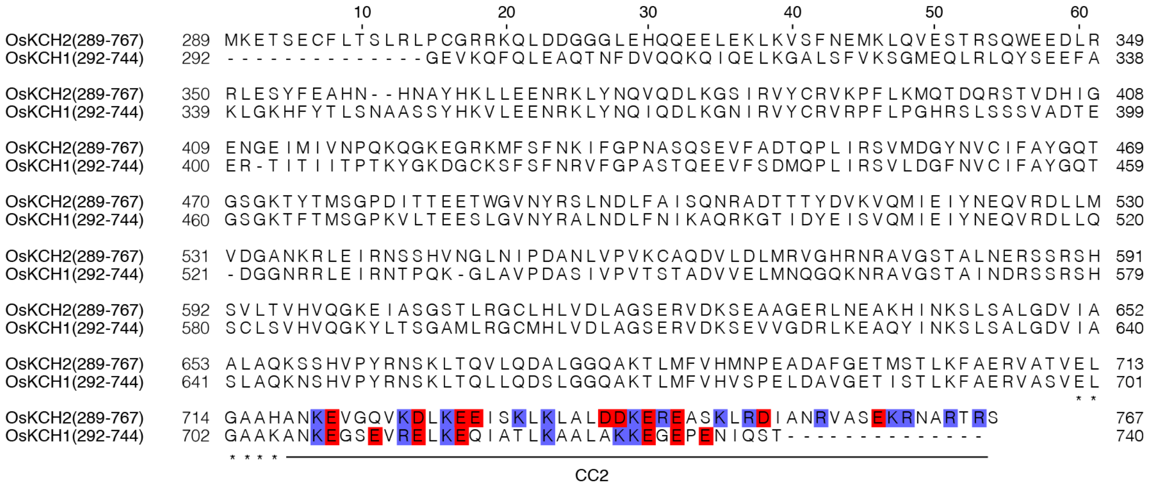

Supplementary Figure 6: OsKCH2(289-767) and OsKCH1(292-744) differ drastically in the CC2 region. Pairwise protein alignment of OsKCH2(289-767) and OsKCH1(292-744). The neck mimic region is indicated by asterisk (*) and the CC2 region is indicated by the underline. Positively charged residues in the CC2 region are highlighted in blue and negatively charged residues in red.

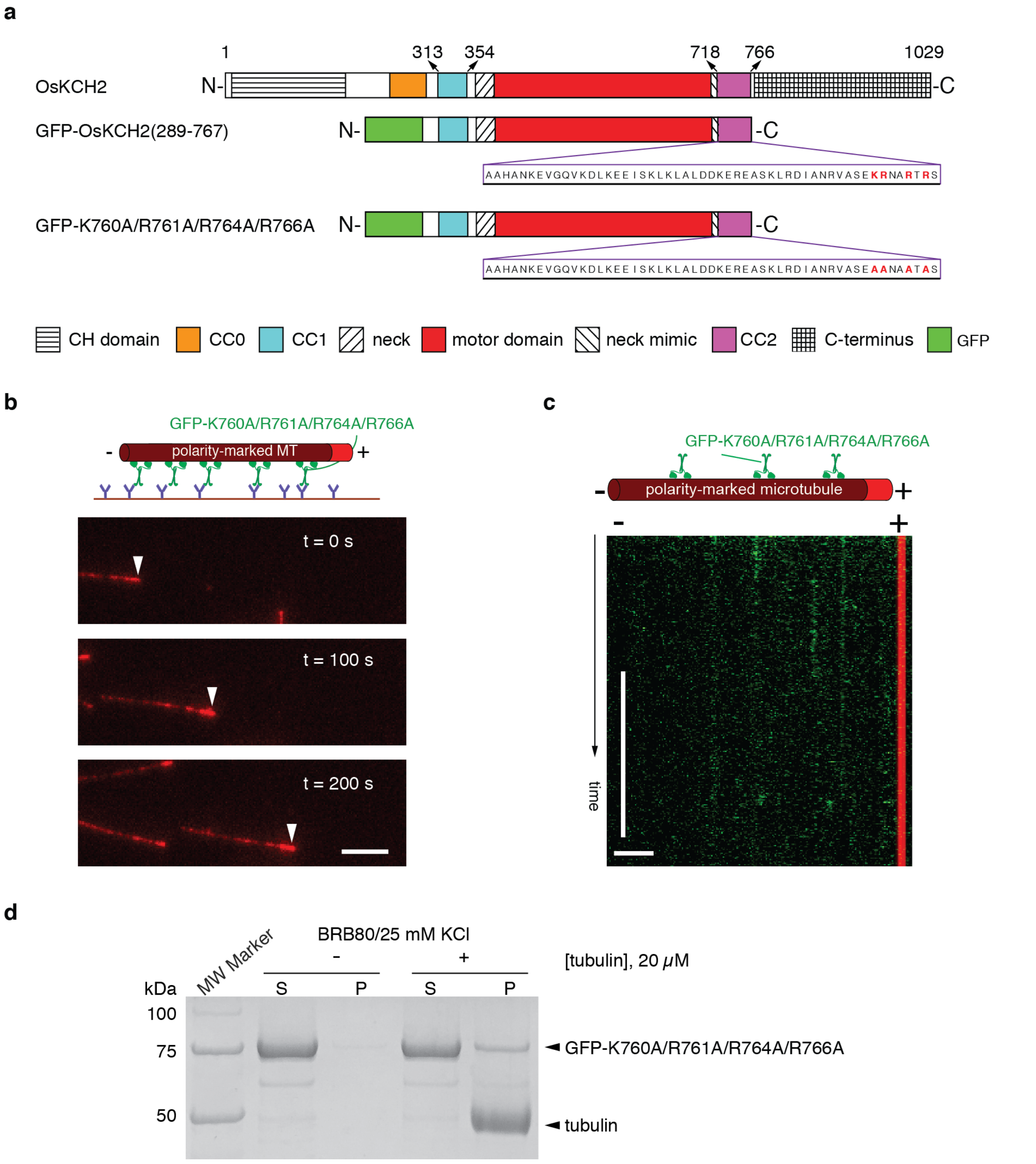

Supplementary Figure 7: The quadruple mutant GFP-K760A/R761A/R764A/R766A is a nonprocessive minus-end-directed motor on single microtubules. a. Schematic diagrams of the full-length OsKCH2, GFP-OsKCH2(289-767) and GFP-

K760A/R761A/R764A/R766A. **b.** Micrograph montage showing that surface-immobilized GFP-K760A/R761A/R764A/R766A molecules drive microtubules to glide with minus-end-directed motility. White arrowheads indicate the plus ends of polarity-marked microtubules. **c.** Example kymograph showing that individual GFP-K760A/R761A/R764A/R766A molecules are unable to produce processive motility on a single microtubule. **d.** Coomassie-stained SDS-PAGE of the microtubule co-sedimentation assay for GFP-K760A/R761A/R764A/R766A in BRB80/25 mM KCl. Scale bars: 30 s (vertical) and 5 μm (horizontal).

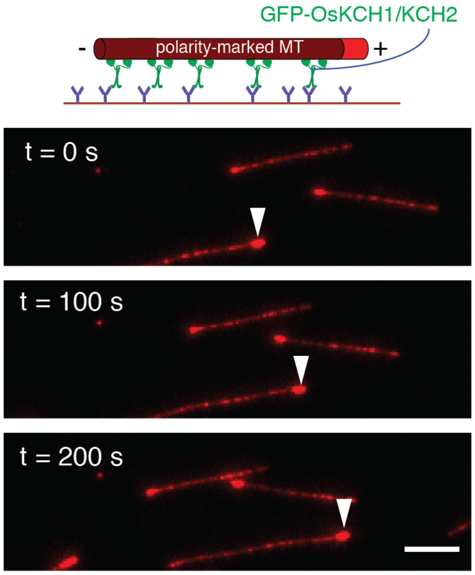

Supplementary Figure 8: GFP-OsKCH1/KCH2 exhibits minus-end-directed motility in the ensemble microtubule-gliding assay. Micrograph montage showing surface-immobilized GFP-OsKCH1/KCH2 molecules drive microtubules to glide with minus-end-directed motility. White arrowheads indicate the plus ends of polarity-marked microtubules.

Supplementary References:

1. Phillips, R. K., Peter, L. G., Gilbert, S. P. & Rayment, I. Family-specific kinesin structures reveal neck-linker length based on Initiation of the coiled-coil. *J. Biol. Chem.* **291,** 20372–20386 (2016).
2. Jie, J., Löhr, F. & Barbar, E. Interactions of yeast dynein with dynein light chain and dynactin: General implications for intrinsically disordered duplex scaffolds in multiprotein assemblies. *J. Biol. Chem.* **290,** 23863–23874 (2015).
3. Delorenzi, M. & Speed, T. An HMM model for coiled-coil domains and a comparison with PSSM-based predictions. *Bioinformatics* **18,** 617–625 (2002).
