# Supplementary material for: The preprophase band-associated kinesin-14 OsKCH2 is a processive minus-end-directed microtubule motor
Source: Nat Commun. 2018 Mar 14;9:1067. doi: 10.1038/s41467-018-03480-w (PMC5852081; doi:10.1038/s41467-018-03480-w)
Supplement: Supplementary file 3 — Description of Additional Supplementary Files [file 41467_2018_3480_MOESM3_ESM.pdf]

Description of Supplementary Files

Supplementary Movie 1: Transport of rhodamine-labeled AFs (red) by unlabeled OsKCH2(1- 767) along surface-immobilized Alexa 488-labeled polarity-marked single microtubules (green). Arrowhead indicates the microtubule plus end.

Supplementary Movie 2: Gliding of TMR-labeled polarity-marked microtubules (red) by surface-immobilized GFP-OsKCH2(289-767). The bright ends are the microtubule plus ends.

Supplementary Movie 3: Movement of individual GFP-OsKCH2(289-767) molecules (green) on surface-immobilized HiLyte 647-labeled polarity-marked single microtubules (red). Arrowhead indicates the microtubule plus end. This movie corresponds to the kymograph on the left in Figure 2f.

Supplementary Movie 4: Movement of individual GFP-OsKCH2(289-767) molecules (green) on surface-immobilized HiLyte 647-labeled polarity-marked single microtubules (red). Arrowhead indicates the microtubule plus end. This movie corresponds to the kymograph on the right in Figure 2f.

Supplementary Movie 5: Movement of individual GFP-OsKCH2(289-767)^{T} molecules (green) on surface-immobilized HiLyte 647-labeled polarity-marked single microtubules (red). Arrowhead indicates the microtubule plus end.

Supplementary Movie 6: Gliding of TMR-labeled polarity-marked microtubules (red) by surface-immobilized GFP-OsKCH1(292-744). The bright ends are the microtubule plus ends.

Supplementary Movie 7: Nonprocessive movement of individual GFP-OsKCH1(292-744) molecules (green) on surface-immobilized HiLyte 647-labeled polarity-marked single microtubules (red). Arrowhead indicates the microtubule plus end.

Supplementary Movie 8: Gliding of TMR-labeled polarity-marked microtubules (red) by surface-immobilized GFP-OsKCH2(289-720). The bright ends are the microtubule plus ends.

Supplementary Movie 9: Nonprocessive movement of individual GFP-OsKCH2(289-720) molecules (green) on surface-immobilized HiLyte 647-labeled polarity-marked single microtubules (red). Arrowhead indicates the microtubule plus end.

Supplementary Movie 10: Gliding of TMR-labeled polarity-marked microtubules (red) by surface-immobilized GFP-K760A/R761A/R764A/R766A. The bright ends are the microtubule plus ends.

Supplementary Movie 11: Movement of individual GFP-K760A/R761A/R764A/R766A molecules (green) on surface-immobilized HiLyte 647-labeled polarity-marked single microtubules (red). Arrowhead indicates the microtubule plus end.

Supplementary Movie 12: Gliding of TMR-labeled polarity-marked microtubules (red) by surface-immobilized GFP-OsKCH1/KCH2. The bright ends are the microtubule plus ends.

Supplementary Movie 13: Movement of individual GFP-OsKCH1/KCH2 molecules (green) on surface-immobilized HiLyte 647-labeled polarity-marked single microtubules (red). Arrowhead indicates the microtubule plus end.

Supplementary Data 1: **Protein sequence alignment of kinesin-14 motors from *O. sativa* and *A. thaliana*.** Protein sequence data of *O. sativa* were obtained from MSU RGAP Release 7 (http://rice.plantbiology.msu.edu/; "LOC_" was omitted from each locus call) except for OsKCH2, which was not annotated in the release. Protein sequence data of *A. thaliana* were from TAIR10 (http://www.arabidopsis.org/). Alignment was performed using the whole protein sequences.
