# Supplementary material for: The preprophase band-associated kinesin-14 OsKCH2 is a processive minus-end-directed microtubule motor
Source: Nat Commun. 2018 Mar 14;9:1067. doi: 10.1038/s41467-018-03480-w (PMC5852081; doi:10.1038/s41467-018-03480-w)
Supplement: Supplementary file 4 — Supplementary Data 1 [file 41467_2018_3480_MOESM4_ESM.pdf]

```
Os06g11380        ------------------------------------------------------------
AtKCA1            ------------------------------------------------------------
AtKCA2            ------------------------------------------------------------
AtKinesin-12A     ------------------------------------------------------------
At3g10310         ------------------------------------------------------------
Os01g14090        ------------------------------------------------------------
At1g63640         ------------------------------------------------------------
At5g41310         ------------------------------------------------------------
Os02g13580        ------------------------------------------------------------
Os06g36080        ------------------------------------------------------------
Os01g54080        ------------------------------------------------------------
Os05g44560        ------------------------------------------------------------
At1g18410         ------------------------------------------------------------
At1g73860         ------------------------------------------------------------
Os11g44880        ------------------------------------------------------------
OsKCH1            ------------------------------------------------------------
Os03g18980        ------------------------------------------------------------
At2g47500         ------------------------------------------------------------
At1g09170         ------------------------------------------------------------
ATK4              ------------------------------------------------------------
AtKP1             ------------------------------------------------------------
OsKCH2            ------------------------------------------------------------
AtKCBP            MEGQRGSNSSLSSGNGTEVATDVSSCFYVPNPSGTDFDAESSSLPPLLSLCHSSPAP-QV
Os04g57140        -----------MNGGGA--------------SGGDGYDSDGYSFAP--------PTPTTL
At1g55550         ------------------------------------------------------------
Os01g15540        ------------------------------------------------------------
At5g27950         ------------------------------------------------------------
Os11g42800        ------------------------------------------------------------
ATK2              ------------------------------------------------------------
ATK3              ------------------------------------------------------------
ATK5              ------------------------------------------------------------
ATK1              ------------------------------------------------------------
Os03g64415        ------------------------------------------------------------
Os04g53760        ------------------------------------------------------------
Os07g01490        ------------------------------------------------------------
At2g22610         ------------------------------------------------------------
Os03g02290        ------------------------------------------------------------
At1g72250         ------------------------------------------------------------
Os12g42160        ------------------------------------------------------------
At5g27550         ------------------------------------------------------------
Os05g33030        ------------------------------------------------------------


Os06g11380        ------------------------------------------------------------
AtKCA1            ------------------------------------------------------------
AtKCA2            ------------------------------------------------------------
AtKinesin-12A     ------------------------------------------------------------
At3g10310         ------------------------------------------------------------
Os01g14090        ------------------------------------------------------------
At1g63640         ------------------------------------------------------------
At5g41310         ------------------------------------------------------------
Os02g13580        -----------------------------------------MGVAKFSASDLERGQMTAV
Os06g36080        ------------------------------------------------------------
Os01g54080        ------------------------------------------------------------
Os05g44560        ------------------------------------------------------------
At1g18410         ------------------------------------------------------------
At1g73860         ------------------------------------------------------------
Os11g44880        ------------------------------------------------------------
OsKCH1            ------------------------------------------------------------
Os03g18980        ------------------------------------------------------------
At2g47500         ------------------------------------------------------------
At1g09170         ------------------------------------------------------------
ATK4              ------------------------------------------------------------
AtKP1             ------------------------------------------------------------
OsKCH2            ------------------------------------------------------------
AtKCBP            ALSIPAELAAAIPLIDRFQVEAFLRLMQKQIQSAGKRGFFYSKKSSGSNVRERFTFEDML
Os04g57140        SMSIPPELAGAIPLIDRFQVEGFLKAMQKQIHSAGKRGFF-SKKSVGPHVREKFTLEDML
At1g55550         ------------------------------------------------------------
Os01g15540        ------------------------------------------------------------
At5g27950         ------------------------------------------------------------
Os11g42800        ------------------------------------------------------------
```

```
ATK2               ------------------------------------------------------------
ATK3               ------------------------------------------------------------
ATK5               ------------------------------------------------------------
ATK1               ------------------------------------------------------------
Os03g64415         ------------------------------------------------------------
Os04g53760         ------------------------------------------------------------
Os07g01490         ------------------------------------------------------------
At2g22610          ------------------------------------------------------------
Os03g02290         ------------------------------------------------------------
At1g72250          ------------------------------------------------------------
Os12g42160         ------------------------------------------------------------
At5g27550          ------------------------------------------------------------
Os05g33030         ------------------------------------------------------------


Os06g11380         ------------------------------------------------------------
AtKCA1             ------------------------------------------------------------
AtKCA2             ------------------------------------------------------------
AtKinesin-12A      ------------------------------------------------------------
At3g10310          ------------------------------------------------------------
Os01g14090         ------------------------------------------------------------
At1g63640          -------------------------------------------------------MSSHL
At5g41310          -------------------------------------------------------MKNRI
Os02g13580         VNCILALKDRFGSRGGDDHRNPGFLTRCDSEGGRKRVESKLQRMLTSPIMSEPSSPVLGS
Os06g36080         -------------------------------------------MLKQKQRRHHARPLAHV
Os01g54080         ------------------------------------------------------------
Os05g44560         ------------------------------------------------------------
At1g18410          -------------------------------------------------------MLESE
At1g73860          ---------------------------------------------MNPMRDQPGSPYGDS
Os11g44880         -------------------------------------------------------MGSPE
OsKCH1             ---------------------------------------------------------MMA
Os03g18980         --------------------------------------------------------MAEA
At2g47500          -----------------------------------------------------MAATATE
At1g09170          --------------------------------------------------------MATE
ATK4               -----------------------------------------------------MATTSEI
AtKP1              ------------------------------------------------------------
OsKCH2             ------------------------------------------------------------
AtKCBP             CFQKDPIPTSLLKINSDLVSRATKLFHLILKYMGVDSSDRSTPPSLDERIDLVGKLFKKT
Os04g57140         CFQKDPIPTSLLKISSDLVSRSIKLFHVILKYMGIDS---PAIISLDERIELVAKLYKHT
At1g55550          ------------------------------------------------------------
Os01g15540         ------------------------------------------------------------
At5g27950          ------------------------------------------------------------
Os11g42800         ------------------------------------------------------------
ATK2               -------------------------------------------------------MVGEM
ATK3               -------------------------------------------------------MVGAM
ATK5               -------------------------------------------------------MPLRN
ATK1               --------------------------------------------------------MASR
Os03g64415         ----------------------------------------------MPIVGNAGAGSVGA
Os04g53760         -------------------------------------------------------MASSL
Os07g01490         -----------------------------------------------------------M
At2g22610          -------------------------------------------------------MDDVQ
Os03g02290         -----------------------------------------------------MEGEEER
At1g72250          ---------------------------------------------------MEDCCDPLL
Os12g42160         ------------------------------------------------------------
At5g27550          ------------------------------------------------------------
Os05g33030         -------------------------------------------------------MLAFI


Os06g11380         ------------------------------------------------------------
AtKCA1             ------------------------------------------------------------
AtKCA2             ------------------------------------------------------------
AtKinesin-12A      ------------------------------------------------------------
At3g10310          -----------MTTGLHEFNLASRR----------------------AEEAAARRFQ---
Os01g14090         ------MAAEPRRVSFRDGRLASRK----------------------AEEAALRRHQ---
At1g63640          SQDANMNGVYVRSDVSSMLSFDGSE------------------SRESMDDSKKGHQS---
At5g41310          KKGSSMIGVYGRSDGSSSIQSSNGS-----------------ESRESIDDNKQGHQS---
Os02g13580         DPYSPLQVFQLKQGGYADQLGGKYSDLLKSTSLDNAPTQSLLGVFNSILDESIERKNGQI
Os06g36080         FSRARSPPPRLALALSLSSSPPRRR-----RLLGGQTPHHQLFDRVPRLPPSLAMSSAAA
Os01g54080         --------MGTVNGEYEDFDAANRR------------------------------AE---
Os05g44560         --------MGSVDGDFEGLQAADRR-----------------------------------
At1g18410          FQREHAFESATEQELTCPISDNLHE----------------------SVEADDDSVQ---
```

```
At1g73860         TPRSPFSPFSPLSVDDRHRNHADTK-----------------------------------
Os11g44880        GEEAVVATAAVVEDGLRGNGDGGGGGVGEVVGVGRSIDMEWRK----AEEAAIRRYE---
OsKCH1            AAVEEEEMVERMHGWARDMDVASRR----------------------AEEEAMRRYD---
Os03g18980        AALFSLSAAAVVEDVLRQHGCRLSD---------------RDLASRRAEEAAARRNE---
At2g47500         DGGLSFTVASVMEDVLQQHGNGLRD---------------HDLVSRRAEEAASRRYE---
At1g09170         QQDSQLCLATILEDFLKQRNIQVSV--GVDSSSLKKADETFGGRDLPVDPSDLRRYE---
ATK4              NNDLSFSVVSIVEDVLQQHSSRSSD---------------VGLVSRKVEESSLRRYE---
AtKP1             ------------------------------------------------------------
OsKCH2            -------MAAAAEGGRHDQGMALRK----------------------AEESAARRCE---
AtKCBP            LKRVELRDELFAQISKQTRHNPDRQYLIKAWELMYLCASSMPPSKDIGGYLSEYIHNVAH
Os04g57140        LKRSELRDELFAQISKQTRNNPDRAWLIRAWELMYLCASSMPPSKDIGAYLSEYVHYIAH
At1g55550         ------------------------------------------------------------
Os01g15540        ------------------------------------------------------------
At5g27950         ------------------------------------------------------------
Os11g42800        ------------------------------------------------------------
ATK2              TNNGRIRPSFPVKDLTSNEGSEYGG-----------------------------------
ATK3              ANNGRIRSAFPVTNGSKDLTPNSAP-----------------------------------
ATK5              QNRAPLPSPNVKKEALSSIPFDKRR-----------------------KE----------
ATK1              NQNRPPRSPNAKKEGLGGISFDKRR---------------------KVET----------
Os03g64415        KPGSWLPEWKRIVVLLGSWTLGVRE-----------------------------------
Os04g53760        TPRSPYPKKENLGNARRGMGVKPGP-----------------------------------
Os07g01490        STRATRPGMLHQKENAADAQAGKRQ-----------------------------------
At2g22610         IDDTFPVDLNGVTSLCSPEIPSFDF-----------------------------------
Os03g02290        CPGEPTPMDFSWTTGWEKAAADDDE-----------------------------------
At1g72250         ATDASPRPESFSRSAEKDIASRSRTVAMADLDSNCELSNDVDMEQSSPDLMKLEQSSDPV
Os12g42160        -------------MEMEEEGSGRGG-----------------------------------
At5g27550         ------------------------------------------------------------
Os05g33030        FESFFFLLLYFLKSKANPHDLSLAR-----------------------------------


Os06g11380        ------------------------------------------------------------
AtKCA1            ------------------------------------------------------------
AtKCA2            ------------------------------------------------------------
AtKinesin-12A     ------------------------MKKHFTLPRN--------------------------
At3g10310         ------AVQWL-----------KSVVGQLGIPNQPSEKEFISCLRNGMIL---CNAINKI
Os01g14090        ------AATWL-----------ESVIGPFGLSRCPSEQEFVAAVRNGIVL---CKAINKI
At1g63640         ------LVEWL-----------NETLPYLKLPWEASEDELRACLRDGTVL---CSLLNQL
At5g41310         ------LVEWL-----------NETLPYLNLPWEASEEELRACLVDGTVL---CNLLNQL
Os02g13580        PYRIACLLRKVILEIERRISTQAGHIRNQNNLIKAREEKYQSRIRVLEVL---AGGMEKD
Os06g36080        DRRRAEAVAWLRALLRGCG---GGGGGQPLPPPHASEDDLRAALADGALL---CAALRRL
Os01g54080        ------VIDWL-----------GGLLPEFDLPLDSSDEELRDYLINGEAL---CYVADKL
Os05g44560        ----AEVIEWL-----------NALLPEYCLPLDSSDDELRELLSDGTVL---CHIVNAL
At1g18410         ------MLDNLTLNTNPAESCESEEIQTKALPSSSSGQDLVASDEDS------EDVELGD
At1g73860         --------------TPRSPFSPFSPLSGDERHKSLAESKFQQALASSGQLDPLSPGSMHH
Os11g44880        ------AANWLRRV--------VGVVCGKDLAEEPSEEEFRLGLRNGIVL---CNALNKV
OsKCH1            ------AASWLRST--------VGVVCARDLPDEPSEEEFRLGLRNGIVL---CNALNKI
Os03g18980        ------AAGWLRRT--------VGAVAARDLPEEPSEEEFRLGLRNGQIL---CGALNRV
At2g47500         ------AANWLRRM--------VGVVGAKDLPAEPTEEGLRLGLRSGIIL---CKVLNKV
At1g09170         ------AARWVRNT--------LGVVGGRDLPADPSEEDFRIALRSGILL---CNVLNRV
ATK4              ------AAGWLRDM--------IGVSNGKDFPGEPSEEEFRLGLRSGIVL---CNVLNKV
AtKP1             ----------------------MDQGAMETLPEKPSEDEFSLALRNGLIL---CNVLNKV
OsKCH2            ------AARWLRQ---------MEAAAAESLPERPSEEEFCVALRNGLVL---CNVLNHV
AtKCBP            DATIEPDAQVLAVNTLKALKRSIKAGPRHTTPGREEIEALLTGRKLTTIVFFLDETFEEI
Os04g57140        GATTDSDVRVLALNTLNALKRSVKAGPRVTIPAREEIEALLSSRKLTTIVFFLDETFEEI
At1g55550         ------------------------MERTRSKPVRNLPETIHSLL----------------
Os01g15540        ----------------------MDVQPARTM-----------------------------
At5g27950         --------------------------------------MLISAEKGEILI----------
Os11g42800        ------------------------------------------------------------
ATK2              -------------------------------PVEFTREDVETLLHERIKYK---------
ATK3              ----------------------ASTTGSEYGPVEFTREDVETLLNERIKYK---------
ATK5              ----------------------TQGTGRRQVLSTVNRQDANSDVGSTEE----CGKVEFT
ATK1              ----------------------QGGTGRRQAFSAVNKQDVTMNSDVGSIEE--CGKVDFT
Os03g64415        ---------------------NEKGSGSRLIKWNGSGSREQKICKTTLPLFQACIKHLNS
Os04g53760        ----------------------RRNVLSAINNGGGTNSDTASVDGGEGGAGPAAPVIEFT
Os07g01490        --------------------------------RTAAGSAARAPLSANAAPPAPDPAIEFA
At2g22610         ----------------------VSDETEKLEIGDTSIDDCDDALGDSMV----CDPNSRL
Os03g02290        ----------------------AESAPAPAPPAPSPQEAAESMI---------------L
At1g72250         ALDGKVVLGFSLASPDL-----VNCGASPDLPRGSYEDSPEFSKKRR------FSTELSL
Os12g42160        ----------------------DGPAAHGRIGGDSPVMVSSASVRKTVKMSETCDFIPYV
At5g27550         ----------------------------------MERDQHQEICNDGGLL---CES----
Os05g33030        -----------------QIHAVDLISRLVSMTRQPSGRPFPNETGKSEMS---CTGA---
```

```
Os06g11380        ------------------------------------------------------------
AtKCA1            ------------------------------------------------------------
AtKCA2            ------------------------------------------------------------
AtKinesin-12A     ----------------AIL-----------------------------------------
At3g10310         HPGAVSKVVENY----SYL-----------------------------------------
Os01g14090        QPGAVPKVV-------ANA-----------------------------------------
At1g63640         SPGSM-------------------------------------------------------
At5g41310         SPGSM-------------------------------------------------------
Os02g13580        KFGDKGQLAVEDME--RLM-----------------------------------------
Os06g36080        GCDPAA----------ASD-----------------------------------------
Os01g54080        MPGV------------LEG-----------------------------------------
Os05g44560        IPG-------------VLE-----------------------------------------
At1g18410         TFYSCSELLQRNCCVLPYK-----------------------------------------
At1g73860         GGHKFHEVFQMKQGRYDLQ-----------------------------------------
Os11g44880        QPGSVPKVVEA-----PSD-----------------------------------------
OsKCH1            QPGAIPKVVQAQSDAAG-------------------------------------------
Os03g18980        HPGAVPKVVVNTAAD-SVL-----------------------------------------
At2g47500         QPGAVSKVVESPCD--AIL-----------------------------------------
At1g09170         KPGAVPKVVEAPND--PLV-----------------------------------------
ATK4              NPGSVSKVVEAPDD----------------------------------------------
AtKP1             NPGSVLKVVENPITP-AIQ-----------------------------------------
OsKCH2            NPGAVPKVVENPIV--AVQ-----------------------------------------
AtKCBP            SYDMATTVSDAVEELAGTIKLSAFSSFSLFECRKVVSSSKSSDPGNEEYIGLDDNKYIGD
Os04g57140        TYDMATTVADAVEELAGIIKLSVYSSFSLFECRKVVNGSKSSDVGNEEYIGLDDNKYIGD
At1g55550         ----------------GLK-----------------------------------------
Os01g15540        ------------------------------------------------------------
At5g27950         ----------------PQS-----------------------------------------
Os11g42800        ------------------------------------------------------------
ATK2              ----------------SKY-----------------------------------------
ATK3              ----------------SKF-----------------------------------------
ATK5              KDEVLALLNERAKA--GKF-----------------------------------------
ATK1              KDEILALLSERAKA--GKF-----------------------------------------
Os03g64415        R---------------DLL-----------------------------------------
Os04g53760        GREDVERLLAEKMKGKSKT-----------------------------------------
Os07g01490        GRDDVDALLNEKMKGKNKM-----------------------------------------
At2g22610         VP--------------TGL-----------------------------------------
Os03g02290        VPGPRVVLS-------GLM-----------------------------------------
At1g72250         ENGIDGSTTTTRLGRKSQV-----------------------------------------
Os12g42160        DDDDDGNSEEENSASSGIL-----------------------------------------
At5g27550         ----------------KEV-----------------------------------------
Os05g33030        ------------------------------------------------------------

Os06g11380        ----------------------MAD-----------------------------------
AtKCA1            ----------MADQRS--------------------------------------------
AtKCA2            ----------MAEQK---------------------------------------------
AtKinesin-12A     ----------RDGGEPHSPNPSISK-----------------------------------
At3g10310         ----------NGEYQLPPAYQYFEN--VRNFLVALETLRLPGFEASDLEKDNLESGSVTK
Os01g14090        ----------SCDSQPSTAFQYFEN--IRNFLVAVQELKLPCFEASDLEKDNIDAGSVGK
At1g63640         ----------RMGGSFEPASVKIER-----FLTAMDEMALPRFEVSDIEQ-----GDMVP
At5g41310         ----------RMGGSFEPGCVNIER-----FLAAMDEMTLPRFESLKALKASFSDDGYDK
Os02g13580        ----------KYQDDVVRLMKENED--LVRLLREKEDMVRLLKEKEDMVRLLKEKEGMIN
Os06g36080        ----------EGTGSAAAAAAAAGEGDVGRFLAAAERMGLPGFSPSDLDT-----GPVSS
Os01g54080        ----------TWGGYASDQRSNVKK-----FLSVVAEMGLPGFGVKDLEE-----GSMSS
Os05g44560        ----------ESWGAYASSDQHAGH--VKKFLAVVADMGLPGFSVKDLEE-----GSMSG
At1g18410         ----------AQKKTKENPFDFDVR-------TWSSPCDFPRFGEMILLSPVLNPMPFFS
At1g73860         ----------ASKISEMMKSSSLDNAPTQSLLSVLNGILDESIERKNGEIPQRVACLLRK
Os11g44880        ----------SADGAALCAYQYFEN--VRNFLMGLQDLGLPTFEASDLEK----GGKGVR
OsKCH1            ----------PTDGSALCAYQYFEN--LRNFLVVVEDLRLPTFEVSDLEK----GGKGVR
Os03g18980        ----------QPDGAALSAFQYFEN--VRNFLVAAQEIGLPCFEASDLEQ----GGKSAR
At2g47500         ----------VADGAPLSAFQYFEN--VRNFLVAIQEMGFPTFEASDLEQ----GGNASR
At1g09170         ----------NQDGAALSAFQYFEN--LRNFLVFVEEMGIPTFEVSDFEK----GGKSAR
ATK4              ----------VADGAALSAFQYFEN--IRNFLVAIEEMGLPSFEASDMEK----GGKSIR
AtKP1             ----------YADGAAQSAIQYFEN--MRNFLKAVEDMQLLTFGASDLEK----GGSSNK
OsKCH2            ----------SSDVAAQSAIQYFEN--MRNFLVAVSEMNLLTFEASDIEK----GGSSMK
AtKCBP            LLAEFKAIKDRNKGEILHCKLVFKKKLFRESDEAVTDLMFVQLSYVQLQHDYLLGNYPVG
Os04g57140        LLSEFKAAKDRNKGEILHCKLVFKKRLFRESDEAITDPMFVQLSYVQLQHDYILGNYPVG
At1g55550         ------------------------------------------------------------
Os01g15540        ----------RNLPDTLSSLMGFNK-----------------------------------
```

```
At5g27950          ----------PLSSESVVPLVYTDV-----------------------------------
Os11g42800         -----------MEGHVIVP-----------------------------------------
ATK2               ----------NYKERCENTMDYVKR--LRLCIRWFQELELDYAFEQEKLKNAM-EMNEKH
ATK3               ----------NYKERCENMMDYIKR--LRLCIRWFQELELDYAFEQEKLKNAL-ELNEKH
ATK5               ----------DTKGKIEQMTDIIKK--LKVCVRWYQQVDETHVQDKENLSSSL-QSAEKR
ATK1               ----------DTKAKIEQMTDIIKR--LKVCVKWFQQADETHVQEKENLKVSL-ESSEQK
Os03g64415         ----------STKVLTLALYKYILR-------APQTAPHPPAAPPRTEVASSTAGRASR
Os04g53760         ----------DYKGRTEQMSEYIKK--LRACIRWYIELEDGYLVEQEKLRSTMDAENAQH
Os07g01490         ----------DYKGKSEQMMEYIKK--LRACIKWLLEREDTNLAEIGKLNGLL-EAAEKH
At2g22610          ----------TRTNRTDETIMFINA-----------------------------GGDDSK
Os03g02290         ----------RGDCRADDSVLFINA-----------------------------GGSATE
At1g72250          ----------VKFSAICQTFGYELS------PESSFELPSPPGDFRESMTPVISINSGSI
Os12g42160         ----------PCDGMQHDTPDYIRR-----------------------------GAAAAR
At5g27550          ----------SVNNHNSDA-----------------------------------------
Os05g33030         ----------PSNGRLVSASSKIKS-----------------------------APPISP


Os06g11380         ---------------------------TRGRWAWDVPGFEPP------------------
AtKCA1             ---------------------------KTNRWNWEVSGFEPR------------------
AtKCA2             ---------------------------STNMWNWEVTGFESK------------------
AtKinesin-12A      ------------------------------------------------------------
At3g10310          VVDCILG--------------------LKAYHECKLPSNGNG------------------
Os01g14090         IVDCVIS--------------------LKSYHEWRQRGGSYG------------------
At1g63640          VLQSLKA--------------------LKASFSDGSYDKNSL------------------
At5g41310          NT-------------------------LSARRRWSLPADHSK------------------
Os02g13580         LKTVKAEETQRIEDEDKYRIIKEKDDALDRLVKEKEEMIRLLKEKDDVVRLMKEKEDLLN
Os06g36080         VVTCLLA--------------------LRDQFVSHDVGGLSC------------SLPEKV
Os01g54080         IVECLLA--------------------LKDNVATQLGGHISN------------------
Os05g44560         VVDCLLV--------------------LRESVSSGLRDGTSK------------------
At1g18410          CCTKRAI--------------------FCSSPGSSHGGSTPR------------------
At1g73860          VVQEIERR-------------------ISTQAEHLRTQNNIF------------------
Os11g44880         VVDCVLS--------------------LRSFSESKQVGRSAP------------------
OsKCH1             VVDCVLA--------------------LKSFSESNKTGRQAS------------------
Os03g18980         VVNCVLA--------------------LKSYGDWKQCGGTGP------------------
At2g47500          VVNCVLA--------------------IKSYDEWKQSGGIGV------------------
At1g09170          IVECVLA--------------------LKSYREWKQSGGSGT------------------
ATK4               IVNCILA--------------------LKSYSEWKLKGENGP------------------
AtKP1              VVDCILC--------------------LKGFYEWKQAGGVGV------------------
OsKCH2             VVDCILC--------------------LKAYHEWKLSGGIGI------------------
AtKCBP             RDDAAQLCALQILVGIGFVNSPESCIDWTSLLERFLPRQIAI------------------
Os04g57140         RDDAAQLSALQILVEIGFVDNPESCVEWISLLERFLPRQVAI------------------
At1g55550          ---------------------------SHMTSDWVKSVCNIA------------------
Os01g15540         ----------------------------HLTPSWIESVSHII------------------
At5g27950          ------------------------------------------------------------
Os11g42800         ---------------------------LEKLSLELNNGGIML------------------
ATK2               CADLEVN--------------------LKVKEEELNMVIDEL------------------
ATK3               CVDMEVS--------------------LKNKEEELNMIIEEL------------------
ATK5               YSDKELD--------------------AKTKEEELRATITEM------------------
ATK1               YNHKELE--------------------ARTKEEELQATISKL------------------
Os03g64415         ---------------------------TKSASSTGRVSRANP------------------
Os04g53760         AKLEAQ---------------------LSSDLEELKAAHLNL------------------
Os07g01490         HSEIVAQ--------------------LKSAIEESKAINEEL------------------
At2g22610          VLDSE----------------------LNISRDDYFEGGDVL------------------
Os03g02290         GCEPS----------------------SKLSEDSFFEGGDAI------------------
At1g72250          STDVTVED-------------------VTFLKDEFFSGGESI------------------
Os12g42160         ---------------------------HRIAPLELFSGPSPP------------------
At5g27550          ---------------------------VEESEDTITSGNQEV------------------
Os05g33030         LPQTPPR--------------------LLLRSPNQIPNSAPP------------------


Os06g11380         ---------------------QPVVGAAAGMPLAPPTA----------------------
AtKCA1             -----------------------KSSSNASFAES--------------------------
AtKCA2             ------------------------------------------------------------
AtKinesin-12A      ------------------------------------------------------------
At3g10310          ---------------LYKHVKTPTFQLSATKIHPTLSA----------------------
Os01g14090         ---------------HLKHLKSPLATRSASHV----------------------------
At1g63640          ---------AARRRWSLPEDHSDSRGDDRNFTDGFQSK----------------------
At5g41310          ------------------------------------------------------------
Os02g13580         LEKGEVEGTTKMTDDNKDRLIKEKNDVVLRLTEEKEEMVRLLKEKEDIIRLMKEKEDMVY
Os06g36080         MMQSMEFPRKENDPGTQNSEGRRKIPKNPAMSEPSSPL----------------------
Os01g54080         ---------STAKTPIRRKLELRETDGPVLSVATPGKR----------------------
```

```
Os05g44560          --------APLRKKWRVPETGEPLVPGVAQGKTSPGED----------------------
At1g18410           ----SPFSPSSPRERHNKGLADSRFQRPLPNSSALDPS----------------------
At1g73860           ------KTREEKYQSRINVLEALASGTGVEHEIATQQL----------------------
Os11g44880          --------------LKYGGILKPSMSGKHFIRKNSEP-----------------------
OsKCH1              --------------CKYGGLSKPLTARKYFILKNTDAF----------------------
Os03g18980          --------------WKYGGNLKPSASGKSFVRKNSEPF----------------------
At2g47500           --------------WKFGGNIKPPALGKSSFVRKNSEP----------------------
At1g09170           --------------WRYILNSKPTTFGIAKQYKRKDSE----------------------
ATK4                --------------WRYGSNMKHNFGSRKLFLRKSSEP----------------------
AtKP1               --------------WRYGGTVRIVSFNRKGSS----------------------------
OsKCH2              --------------WRYGGIVKIASSSKRLPSYSSRGG----------------------
AtKCBP              --------TRAKREWELDILARYRSMENVTKDDARQQF----------------------
Os04g57140          --------TRAKRDWELDIVSRYQLMEHLSKDDARQQF----------------------
At1g55550           ------------------------------------------------------------
Os01g15540          ------------------------------------------------------------
At5g27950           ------------------------------------------------------------
Os11g42800          ------------------------------------------------------------
ATK2                ------RKNFASVQVQLAKEQTEKLAANESLGKEREAR----------------------
ATK3                ------RKNFESVQVQLAREQTEKLAANDSLGKEKEAR----------------------
ATK5                ------KENIESLQEKLSKEKLSKLDAIENHRREKDCR----------------------
ATK1                ------EENVVSLHEKLAKEESSTQDAIECHRREKEAR----------------------
Os03g64415          -----------------ASFHRPRLLRQAGLHLRRPRL----------------------
Os04g53760          ------TRQCDSLEESFNKEKADRMLAVESYEKERQQR----------------------
Os07g01490          ------QRQYASLEENLKRVEAEKLDALRSYGDEKEAR----------------------
At2g22610           -------RTEESIVEAGDFPFIYQSARVGNFCYQLNNL----------------------
Os03g02290          -------ETSEDIVEGGDYPSLYHSARYGNFSYKIDGL----------------------
At1g72250           --------TTDAVVGNEDEILLYQTARLGNFAYKFQSL----------------------
Os12g42160          ---------------QGPPSPSPAIGGAALEATSNDGV----------------------
At5g27550           ------------------------------------------------------------
Os05g33030          ---------RARRRKQIAAMEADPAPSSTPPPSSPAPA----------------------


Os06g11380          ------------------------------------------------------------
AtKCA1              ------------------------------------------------------------
AtKCA2              --------------------------------------------------KSPSSEEGVH
AtKinesin-12A       ------------------------------------------------------------
At3g10310           -----------------------------SKTSRHLDMSSVRERNDCTDGESDKLKGIAK
Os01g14090          --------------------QSEYVCSGSSSTPKRLDLVETDTERQPNQNVGPNCQEAME
At1g63640           ----------------------EGSEIDMSDAKISDLLKSNSLRNAPTRSLFDMLDKLLD
At5g41310           ------------------------GVDSNFNDGGSQFIEASEINTSHHSLQNTSTRSLFD
Os02g13580          LEKGEVEDRKQMTDDIKDKLIKEKDDIVFRLTKEKEEIIKLLEEKEDIIILMKEKEDMVN
Os06g36080          --------------------SQTTLSSISRHAGHSFHDVFQLRQGRYSDLPSSKISEMMK
Os01g54080          --------------------YPKSQQRSPLLSGQKINEVVQFKHGTYTDLPAAKISEMLH
Os05g44560          --------------KRNGLPDPKSQQKTPIFNGRKLREIFQLKRGSYADLPAAKISEMMH
At1g18410           --------------------SPGSMLHGGHKSHEAFQMKQG-----RFDLQAAKISELMK
At1g73860           -----------------RQIETEKSMWEEKKKHEEEDMVKLMKQNDQHNLEISALKQELE
Os11g44880          --------------------FVKTMVRSYSAELLRDGVSLEQSLGLDFSLEHVERTTPDS
OsKCH1              ---------------------MNKIMKGHSAEAIQSEFSEGQSIVTDFSIESNEMTTSDS
Os03g18980          --------------------------RRCQSMNEGEVPYEEAGFSGDYHLDSGDMSTSRP
At2g47500           ------------------------FMNSLSRTSSINNEKAPSENDSNKLSSPSSLSTLVR
At1g09170           ----------------------VPVDAVTNSPSSTPSSEQPLLDQSDSNTKNDGTASSID
ATK4                ------------------------------FVSSISRTQSTDMLSTDQPLSSDGDSRSIN
AtKP1               --------------------PPQYGIGSESTTDESVSLDESESSQYDQLLDFLHLSNEIS
OsKCH2              -----------------------------GSADLNQQMLEFVHLLSEVSLEESRVGESQH
AtKCBP              ----LRILKALPYGNSVFFSVRKIDDPIGLLPGRIILGINKRGVHFFRPVPKEYLHSAEL
Os04g57140          ----LRILRTLPYGNSVFFSVRKIDDPIGLLPGRIILGINKRGVHFFRPVPKEYLHSAEL
At1g55550           ------------------------------------------------------------
Os01g15540          ------------------------------------------------------------
At5g27950           ------------------------------------------------------------
Os11g42800          ------------------------------------------------------------
ATK2                ------------------------IAVESLQAAITEELAKTQGELQTANQRIQAVNDMYK
ATK3                ------------------------LSVEKAQAGLTEELGKAQGDLQTANQRIQSVNDMYK
ATK5                ------------------------VVAEKLQVSLREELDKVKEEKMAAKQKVTSLEDMYK
ATK1                ------------------------VAAEKVQASLGEELDKVKEEKMAAKQKVTSLEDMYK
Os03g64415          ------------------------SRQAGLRCHRLHLSAPSRSPTPPPNAVAPSSCSRLP
Os04g53760          ------------------------ESAEASLDLLSVDLERVSHEAKRFSEQLKMVQDTNK
Os07g01490          ------------------------IAVEASRNEHLEDLRRIKLEEKRLNDQIKMLQDTNK
At2g22610           --------------------LPGEYLIDFHFAEIINTNGPKGIRVFNVYVQDEKATE-FD
Os03g02290          --------------------APGDYFLDLHFAEIVNTYGPKGIRAFDVLVQEEKILSELD
At1g72250           --------------------DPGDYFIDLHFAEIEFTKGPPG------------VISGLD
Os12g42160          -----------------------AEPQVHPPEGISSIISTGGGEQETATMGSQSVHETLH
```

```
At5g27550          ------------------------------------------------------------
Os05g33030         ------------------------ASPSRHPPGEEGGGAERVEVEEYVDPPSPDCCGGA-


Os06g11380         ------------------------------------------------------------
AtKCA1             ------------------------------------------------------------
AtKCA2             R-----------------------------------------------------------
AtKinesin-12A      ------------------------------------------------------------
At3g10310          LFADHIFSSKEN------------------------------------------------
Os01g14090         R-----------------------------------------------LQKVILDCMISC
At1g63640          ESMTKMNGHVSH-------------------------------AMASLLSALVQVIEQRI
At5g41310          MLDRLLDESSQK--------------------------MNVSHVYVSILRGIVQVVEQRI
Os02g13580         LGKGEDEDRKQMADDNKDRLIKEKDDIVVRLTKEKEEIIKEKDDIVVRLTKEKEEIIKLL
Os06g36080         S-TSLDNAPTQS------LLSVVNVILDELVETKIGEIPY---HLACLLRKVILEIERRI
Os01g54080         S-NSLDNAPTQS------LLRVVNGILDESIERKRGEIPH---RVVHLLRNVIQEIEHRI
Os05g44560         S-NSLDNAPTQS------LLSVVNGILDESIERKKGEIPH---RVVYLLRKVVQEIERRL
At1g18410          S-NNLDNAPTQS------LLSIVNGILDETIERKNGELPQ---RVACLLRKVVQEIERRI
At1g73860          TTKRKYEQQYSQIESQTKTEKSKWEEQKKNEEEDMDKLLKENDQFNLQISALRQELETTR
Os11g44880         I-RMLVQTMLSD--------------------KKPEEIPS---LVESLLSRVIHEFERRT
OsKCH1             LSILLRKVLLDK---------------------KPEEVPL---IVESILSKVIQEYEHRI
Os03g18980         L-KMLVSAVLSD--------------------KRPDEVPQ---LLESMLSKLVEEFENRL
At2g47500          AVLSDKKPEDVP------------------------------KLIESLLSKVVEEFENRV
At1g09170          AIVRAVFSDMKQ-----------------------EDIPV---IVEDMLKSVMVEYERRL
ATK4               GLVRSFIADRKH-----------------------EDIPN---VVESVLNKVMEEVQQRL
AtKP1              A-EESETAISLAFLFDHFALQLLHGYLKES--DGINDMPLNEMVIDTLLNRVVKDFSAIL
OsKCH2             SLFQQFVLRVVR--------------AFLQEWGEAEGLPLDDMVLETILEQACKEFTILL
AtKCBP             RDIMQFGSSNTAVFFKMRVAGVLHIFQFETKQGEEICVALQTHINDVMLRRYSKARSAAN
Os04g57140         RDIMQFGSSNTAVFFKMRVAGVLHIFQFETKQGEEICVALQTHINDVMLRRYSKARSATS
At1g55550          ------------------------------------------------------------
Os01g15540         ------------------------------------------------------------
At5g27950          ------------------------------------------------------------
Os11g42800         ------------------------------------------------------------
ATK2               L-LQEYNSSLQL---------------------YNSKLQGDLDEAHENIKRGEKERTGIV
ATK3               L-LQEYNSSLQL---------------------YNSKLQGDLDEAHETIKRGEKERTAII
ATK5               R-LQEYNTSLQQ---------------------YNTKLQTDLEVAREAHTRAEKEKSSIL
ATK1               R-LQEYNTSLQQ---------------------YNSKLQTDLETVRAALTRAEKEKSSIL
Os03g64415         RARLAIRGRRAS----------------------------------SHSATVDTACSRRH
Os04g53760         R-LQEYNTSLQQ---------------------YNSNLQADASKSGDIISKLQKEKSAMM
Os07g01490         R-LQEYNTSLQQ---------------------YNSNLQADATKNGETIAKLQKEKNTMV
At2g22610          I-FSVVGANRPLLLVDLRVMVMDDGLIRVRFEGINGSPVVCGI----------------C
Os03g02290         V-YAVVGGNRPLQVRDIRVTVESDSAIVINFKGVRGSPMVCGICIRKRVAMAVTDMVTEG
At1g72250          L-FSQVGANTPLVIEDLRMLVGREGELSIRLEGVTGAAILCGISIRKETTATYVEETGML
Os12g42160         I----------------------------------------------------EENEGKC
At5g27550          ------------------------------------------------------------
Os05g33030         ------------------------------------------------------------


Os06g11380         ---------MPRAPPTAMVARAAGADGAV--VPVADRLDQ--------------------
AtKCA1             ---------TGHRTTGPLLRRNSISTPSL--PPKQAIASK--------------------
AtKCA2             ---------TPSSMLRRYSIPKNSLP-----PHSSELASK--------------------
AtKinesin-12A      ------------------------------------------------------------
At3g10310          -IDENLV--SLENGSENSRANFEKILSRF--PELQSVFKN--------------------
Os01g14090         KENLDND--SLKKDPYKLVGTILSRQLEK--EQFEPLLQL--------------------
At1g63640          SNQAD----NLKNQNILFRVREEKYRSRI--KVLESLAAG------------TTKENEIV
At5g41310          SNQAE----NLKNQNILFRVREEKYRSRI--NVLETLASG--------------------
Os02g13580         EEKEDII--SLMKQKEDMFMSIKEKENKA--ELKKIADED--------------------
Os06g36080         STQAE----HIRNQNNLMKAREEKYKSRI--RVLEALASGTSDQTHVNSNATNGKAHVSP
Os01g54080         GIQAD----HIRNQNSIIKTREDKYRSKI--KALETLVNGTNEENEMAIN----------
Os05g44560         CIQAE----HIRSQNVIIKTREDKYHSKI--KALEILVNG------------TNEENQMA
At1g18410          STQSE----HLRTQNSVFKAREEKYQSRI--KVLETLASG--------------------
At1g73860          KAYEQQCSQMESQTMVATTGLESRLKELEQEGKVVNTAKN--------------------
Os11g44880         ANQNE----SVKHALDPNDDKLLSRADTP--PEMESTCTC--------------------
OsKCH1             AIQNK-------------------------------------------------------
Os03g18980         TSQHE----LVKAALKNGTDGTKSFSKSK--VLVEATPNS--------------------
At2g47500          TNQYE----LVRAAPRESTSSQNNRSFL---KPLGERERE--------------------
At1g09170          ATQNELLLMSAGNRDKLGSGDLGRTISGNEETLSDASYGE--------------------
ATK4               SIHNE----MMKSSSKPIPEDDSSCETVVRSQLCDARQHE--------------------
AtKP1              VSQGA----QLGSFLRKILKCDNGDLSRS--EFLAAVFRY--------------------
OsKCH2             ASHRN----QVRSLLRKMMKDENGAHSKQ--ELIEVISKS--------------------
AtKCBP             SLVNGDI--SCSSKPQNFEVYEKRLQDLS--KAYEESQKK--------------------
Os04g57140         AVSQNDV--SQTYKPPNIEIYEKRVQELS--KAVEESERK--------------------
```

```
At1g55550          ------------------------------------------------------------
Os01g15540         ------------------------------------------------------------
At5g27950          ----------------------------------NVVPEH--------------------
Os11g42800         ------------------------------------------------------------
ATK2               E--------SIGNLKGQFKALQDQLAASK--VSQDDVMKQ--------------------
ATK3               E--------NIGNLKGQFSALQEQLAASK--ASQEDIMKQ--------------------
ATK5               E--------NLTTLRGHSKSLQDQLASSR--VSQDEAVKQ--------------------
ATK1               E--------NLSTLRGHSKSLQDQLSSSR--VLQDDAIKQ--------------------
Os03g64415         SCD------CLNRHRHLAVVATSAVAATTVAVPTACLCCG--------------------
Os04g53760         E--------TMASLKDLNNSMKNHLDSSR--TSQQEAIRM--------------------
Os07g01490         E--------TMNGLKDHANSVKMQLDLAK--SSQNEALKQ--------------------
At2g22610          LRKAPQV--SVPRTSQDFIKCENCATEIEISPTRKRLMRA--------------------
Os03g02290         NVLCKRC--SAHTGNSPLQTRTSKLISKYEKQIEELTNQC--------------------
At1g72250          AVKGS----TDTVLSQQTQENLVCRAEEEAEGMRSDCEQQ--------------------
Os12g42160         S--------CCGQLKQEYSLLLREKEECR--RVLEDLMRE--------------------
At5g27550          ---------SPANGPTL--------------PILQKIIDC--------------------
Os05g33030         ---------DPDHAPPPSPKGEEPVVSAE--EEQAAVAGG--------------------


Os06g11380         -----------------------------------------LADSVQLAREDCLELRQEA
AtKCA1             --------------------------------------VNGLKEKVKLAKEDYLELRQEA
AtKCA2             --------------------------------------VQSLKDKVQLAKDDYVGLRQEA
AtKinesin-12A      ------------------------------------------------------------
At3g10310          -------------------------------LLSEGTLKPSDLKSMPLEELPVHEEDQSS
Os01g14090         -----------------------------------------FSPEGVTVKNESCPPIESS
At1g63640          TNCMEHIKLEKTRIEEKERSEEKDVVRLRKEKERSDAEIRQLKQELKLVKETHENQCLEL
At5g41310          -------------TTDENEVRRKRCAPNRKGKERSNAELSKLKQELEIVKETHEKQFLEL
Os02g13580         --------------AARSIKDKAEIMRLMKEKEDGNNTILKLKKESETLRSSYEESCRLL
Os06g36080         DHAVHQMKMEKDKTEDKKRLAEKDVVLLVKDKEEDVTRLTKDKEDMAKLLKDKEDIIRLM
Os01g54080         --RLEVVKVEKSKIDEKRKLGEQDMIRLIREKENAENIIASLHQEMQVMNRMHEQFREQM
Os05g44560         INRLQIIKEEKSKIEEKRKLGEQDVARLMKEKEISENTIASLKKEMEVMTSMHEQQLQKI
At1g18410          --TSEENETEKSKLEEKKKDKEEDMVGIEKENGHYNLEISTLRRELETTKKAYEQQCLQM
At1g73860          ------------------------------ALEERVKELEQMGKEAHSAKNALEEKIKQL
Os11g44880         ---------------------------STGNMDEEDHTSVSMKEEVSTAVLVNGENVVEH
OsKCH1             -------------------------------MDEEEQNLLNITEQVNHVVVNGDGEVKQF
Os03g18980         -----------------------------------------NEKKMDTIEVYSKHRQTKK
At2g47500          ------------------------------------------EKSFKAIKKDDHNSQILD
At1g09170          -------------------------------ENVTEIVNNNMEASQDSNVEELENQDYEL
ATK4               -------------------------------EAEENSPPQVVEKKFQRTNFEHHEE----
AtKP1              -----------------------------------------LQHRKDLVSKEFSKFCKCG
OsKCH2             -----------------------------------------MKETSECFLTSLRLPCGRR
AtKCBP             ---IEKLMDEQQEKNQQEVTLREELEAIHNGLELERRKLLEVTLDRDKLRSLCDEKGTTI
Os04g57140         ---ADLLNEELQKKTKQERDMQKELEGLRDTLQSERQSIKEVTNDLDKLKSLCDEKDSSL
At1g55550          ------------------------------------------------------------
Os01g15540         ---------------------------------------DGLSPTKPQMKVMVEKDENIS
At5g27950          ------------------------------------------------------------
Os11g42800         ------------------------------------------------------------
ATK2               -------------------------------KDELVNEIVSLKVEIQQVKDDRDRHITEI
ATK3               -------------------------------KGELVNEIASLKVELQQVKDDRDRHLVEV
ATK5               -------------------------------KDSLLMEVNNLQSELQQVRDDRDRHVVQS
ATK1               -------------------------------KDSLLSEVTNLRNELQQVRDDRDRQVVQS
Os03g64415         -------------------------------------QIPLGRHRHNQAVTGRDLTVAAL
Os04g53760         -------------------------------KEQLMKEVDCLRIELHQIREDRDQSVSQV
Os07g01490         -------------------------------KTDLLKEVDNLRGELQQVRDDRDHKLAEI
At2g22610          -----------------------------KAHDKYEKKIAELSERYEHKTNECHEAWMSL
Os03g02290         -------------------------NMKSDECYMAWSSVESTNQELERLKIELHQKVMQS
At1g72250          ----------------------------RKEMEDMKRMVEELKLENQQKTRECEEALNSL
Os12g42160         -------------------------------NELKSRECHEAQASLHELRMELMRKSMHV
At5g27550          ------------------------------------------------------------
Os05g33030         -------------------------------------EGEALRSFLEEFGDQGDDSLVPS


Os06g11380         SDLLE-------------------------------------------------------
AtKCA1             TDLQE-------------------------------------------------------
AtKCA2             TDLQE-------------------------------------------------------
AtKinesin-12A      ------------------------------------------------------------
At3g10310          RSLSH-------------------------------------------------------
Os01g14090         NSQVE-------------------------------------------------------
At1g63640          EAKAQ-------------------------------------------------------
At5g41310          KLNAQ-------------------------------------------------------
Os02g13580         ESKKE-------------------------------------------------------
```

```
Os06g36080        KEKEE----MVWMMREKENMVSLNNGRVEDKHQLTDKDVANSAKYRNEIIKLMKEKEDSN
Os01g54080        ETKAR-------------------------------------------------------
Os05g44560        ELTAK-------------------------------------------------------
At1g18410         ESKTKGATAGIEDRVKELEQMRKDASVARKALEERVRELEKMGKEADAVKMNLEEKVKEL
At1g73860         QQMEK-------------------------------------------------------
Os11g44880        IQAKQ-------------------------------------------------------
OsKCH1            QLEAQ-------------------------------------------------------
Os03g18980        EAYGE-------------------------------------------------------
At2g47500         EKMKT-------------------------------------------------------
At1g09170         YAISK-------------------------------------------------------
ATK4              ------------------------------------------------------------
AtKP1             GKLEF-------------------------------------------------------
OsKCH2            KQLDD-------------------------------------------------------
AtKCBP            QSLMS--------------------------------------------ELRGMEARLAK
Os04g57140        QASLM--------------------------------------------EKTRLETRLKS
At1g55550         ------------------------------------------------------------
Os01g15540        DDNTE-------------------------------------------------------
At5g27950         ------------------------------------------------------------
Os11g42800        ------------------------------------------------------------
ATK2              ETLQA-------------------------------------------------------
ATK3              KTLQT-------------------------------------------------------
ATK5              QKLAG-------------------------------------------------------
ATK1              QKLSE-------------------------------------------------------
Os03g64415        VLLAE-------------------------------------------------------
Os04g53760        NTLSA-------------------------------------------------------
Os07g01490        HSLLA-------------------------------------------------------
At2g22610         TSANE-------------------------------------------------------
Os03g02290        DNIEQ-------------------------------------------------------
At1g72250         SEIQN-------------------------------------------------------
Os12g42160        GSLAF-------------------------------------------------------
At5g27550         ------------------------------------------------------------
Os05g33030        PKLKQ-------------------------------------------------------


Os06g11380        ------------------------------------------------------------
AtKCA1            ------------------------------------------------------------
AtKCA2            ------------------------------------------------------------
AtKinesin-12A     ------------------------------------------------------------
At3g10310         ------------------------------------------------------------
Os01g14090        ------------------------------------------------------------
At1g63640         ------------------------------------------------------------
At5g41310         ------------------------------------------------------------
Os02g13580        -----------------------------DVARLLTDKENNDSIISELKKELEETKRLHE
Os06g36080        DTIMKLNIELEAMKSSYEGTRILLDSKKKEVLQLLMDKESIEYIVSQLKQELAIERSSHQ
Os01g54080        ------------------------------------------------------------
Os05g44560        ------------------------------------------------------------
At1g18410         QKYKDETITVTTSIEGKNRELEQFKQETMTVTTSLEAQNRELEQAIKETMTVNTSLEAKN
At1g73860         ------------------------------------------------------------
Os11g44880        ------------------------------------------------------------
OsKCH1            ------------------------------------------------------------
Os03g18980        ------------------------------------------------------------
At2g47500         ------------------------------------------------------------
At1g09170         ------------------------------------------------------------
ATK4              ------------------------------------------------------------
AtKP1             ------------------------------------------------------------
OsKCH2            ------------------------------------------------------------
AtKCBP            SGNTKSS-----------KETKSELAEMNNQI--LYKIQKELEVRNKELHVAVDNSKRLL
Os04g57140        GQGQESSNRTGVSGNHFERDTLPTVGTVNNSIEMLAKLEEELKSCKKELDASKELSKKLT
At1g55550         ------------------------------------------------------------
Os01g15540        ------------------------------------------------------------
At5g27950         ------------------------------------------------------------
Os11g42800        ------------------------------------------------------------
ATK2              ------------------------------------------------------------
ATK3              ------------------------------------------------------------
ATK5              ------------------------------------------------------------
ATK1              ------------------------------------------------------------
Os03g64415        ------------------------------------------------------------
Os04g53760        ----------------------------------------------------------EL
Os07g01490        ------------------------------------------------------------
At2g22610         ------------------------------------------------------------
Os03g02290        ------------------------------------------------------------
```

```
At1g72250        ------------------------------------------------------------
Os12g42160       ------------------------------------------------------------
At5g27550        ------------------------------------------------------------
Os05g33030       ------------------------------------------------------------


Os06g11380       ------------------------------------------------------YSNAKL
AtKCA1           ------------------------------------------------------YSNAKL
AtKCA2           ------------------------------------------------------YSNAKL
AtKinesin-12A    -----------------------------------------------------SKPPRKL
At3g10310        --------------------------------------------------KTKCNHKRLL
Os01g14090       ------------------------------------------------------NRRRLL
At1g63640        -----------KTRDELEKKLKDAELHVVDSSRKVKELEKLCQSKSQRWEKKECIYQNFI
At5g41310        ---------------KAKVELERQVKNSELRVVEAKELEKLCETKTKRWEKKEQTYKRFI
Os02g13580       AHSQQLETKAAQVSKELEQRIEEVKLMLDDSTKRRIELEELSETRIQFWKKKEVVIDQFV
Os06g36080       THIQELETRAFQANNKLEQRIKEMELMLEDSKTRVRDLEELLESRSQIWEQKEIRLNQFI
Os01g54080       -----------QMEEHLTLRAKEAEFCLMQSKKKVEEVEATSQLKSQLWSKKANIFQSFM
Os05g44560       -----------QMEEHLTTKIKEVESLLVQSNKKIEEVEAASLLKSQLWNKKEGIFQKYM
At1g18410        RELEQSKKETMTVNTSLKAKNRELEQNLVHWKSKAKEMEEKSELKNRSWSQKELSYRSFI
At1g73860        --------ETKTANTSLEGKIQELEQNLVMWKTKVREMEKKSESNHQRWSQKELSYKSFI
Os11g44880       -------------------------------------------------------TDKYF
OsKCH1           ---------------------------------------------------------TNF
Os03g18980       ---------------------------------------------------VTLKQYSML
At2g47500        ---------------------------------------------------RQFKQLTIF
At1g09170        --------------------------------------------------EKTEKQQLII
ATK4             --------------------------------------------------------QKIL
AtKP1            --------------------------------------------SRLNAREFSPGHVEAI
OsKCH2           --------------------------------------------------------GGGL
AtKCBP           SENKILEQNLN-IEKKKKEEVEIHQKRYEQEKKVLKLRVSELENKLEVLAQDLDSAESTI
Os04g57140       MENNLLDQKVQRLERAKSEEKSNMERVYEDECCKLKSRIAELEQKLESRTRSLNVTESTL
At1g55550        -------------------------------------------------------KNTSS
Os01g15540       -------------------------------------------------------SEAKV
At5g27950        ---------------------------------------------------ESNQLEKSI
Os11g42800       ------------------------------------------------------NHDKDI
ATK2             ----------------------------------EATKQNDFKDTINELESKCSVQNKEI
ATK3             ----------------------------------EATKYNDFKDAITELETTCSSQSTQI
ATK5             -----------------------EILMYKESVGKSSHELDILIAKSGSLEETCSLQKERI
ATK1             -----------------------EIRKYQENVGKSSQELDILTAKSGSLEETCSLQKERL
Os03g64415       -------------------------------VARETTGYSNGAPDPAAVVAISCCRPGSI
Os04g53760       ANYKELAGKSTKDCESLSVKVSAFEVSGFRLTKSILSVRNYLAHHYASAQETCSMQQEQI
Os07g01490       -----------------------DVSTYKEMTGKSVAELDNAMTRSTALEETCSSQAERI
At2g22610        -----QLEKVMMELNNKIYQARSLDQTVITQADCLKSITRKYENDKRHWATAIDSLQEKI
Os03g02290       --------------------------VVDRQADQLRSVSQKYENAKKLWAAAISNLENKI
At1g72250        -----------------------ELMRKSMHVGSLAFAVEGQVKEKSRWFSSLRDLTRKL
Os12g42160       -------------------------------------AVEGQVKEKSRWCQLLNDLSEKF
At5g27550        --------------------------------------------------------SDKI
Os05g33030       ------------------------------------------------------------


Os06g11380       GRVTRYLGFLADRTRKLDQ-------------------------AALETEARITPLIHEK
AtKCA1           DRVTRYLGVLAEKSRKLDQ-------------------------FVLETEARISPLINEK
AtKCA2           ERVTRYLGVLADKSRKLDQ-------------------------YALETEARISPLINEK
AtKinesin-12A    RSAKENAPPLDRNTSTPDHRSMRMKNPLPPRPPPSNPLKRKLS-AETATESGFSD-----
At3g10310        KTQEKELAVLKNLFIKTKQ---DFKEFQVYLQRDLMELGNQMQ-EMSSAAQGYYKVVEEN
Os01g14090       QAQESELLELKSMFQEVKI---DFRTLKTQFQDDIIKLGDNVQ-GLSKAALGYNQAVKEN
At1g63640        DNHSGALQELSATSLSIKH---EVVRTQRKYFEDLNYYGLKLK-GVADAAKNYHVVLEEN
At5g41310        NHQTEALQELKATSMSLKH---DVLKIGENYFLDLTYYGIKLR-GVAHAAKNYQIIIEEN
Os02g13580       SLQVQNVQDLKLSSVSVRH---EILNCQNKWSEELAGLGKSLK-VVTNTAEKYHGALAEN
Os06g36080       GLQIQNIQDLRLSSVSIRH---EILHCQKRWSEEICDLGQSLK-VLTNAAENYHATLEEN
Os01g54080       NNQKLSIKDIKISSQSIKQ---EMYALQMTWRDEISNIGHDLK-GLVDAAENYHKVLAEN
Os05g44560       NSQQLYVKGLRISSWSIKN---EMHALEMELRDEMSNFGSGLK-CLVDAAENYHKVLAEN
At1g18410        SFQCQALQELRFYSKSIKQ---EILKVQDKYTVEFSQLGKKLL-ELGDAAANYHEVLTEN
At1g73860        DNQSQALLELRSYSRSIKQ---EILKVQENYTDQFSQLGKKLI-ELSNAAENYHAVLTEN
Os11g44880       DQQQKHIKDLKSNLATMKS---GMEHIKLQYSEDLDKLGKHVH-TLSHAASGYHKVLEEN
OsKCH1           DVQQKQIQELKGALSFVKS---GMEQLRLQYSEEFAKLGKHFY-TLSNAASSYHKVLEEN
Os03g18980       QLQSKHVEELKADIRATKA---GMEFMQMKYSEDINILGRHLF-SLAHAASGYHIVLEEN
At2g47500        NQQQEDIEGLRQTLYTTRA---GMQFMQKKFQEEFSSLGMHVH-GLAHAASGYHRVLEEN
At1g09170        ERQQTHTEELKHDLKAVKA---GLSLLQMKYQQEFTSLGKHLH-GLTYAATGYQRVLEEN
ATK4             LNQQKHIQELKQTLYTTKA---GMKLLQMKYQEDFFHLGKHLN-GLAYAATGYKRVLEEN
AtKP1            GLQQKELEEVKSNFVETRS---QVKQMQSEWQKELQRIVHHVK-AMEVTSSSYHKVLEEN
OsKCH2           EHQQEELEKLKVSFNEMKL---QVESTRSQWEEDLRRLESYFE---AHNHNAYHKLLEEN
```

```
AtKCBP               ESKNSDMLLLQNNLKELEE-LREMKEDIDRKNEQTAAILKMQGAQLAELEILYKEEQVLR
Os04g57140           ALRNAEVDTLQNSLKELDE-LREFKADVDRKNQQTAEILKRQGAQLIELENLYKQEQVLR
At1g55550            TSKKEEDDFVSVDLQSIRD-------------------------QLSALTVQVNDQNKLR
Os01g15540           QKIQDELVSLNAQLKQITL---------------------------------------QR
At5g27950            SNLEEEVFELKLKLKSLDE---------------------------------------KR
Os11g42800           SALQEEISALRSRQRHLDH---------------------------------------RR
ATK2                 EELQDQLVASERKLQVADL---STFEKMNEFEEQKESIMELKG-RLEEAELKLIEGEKLR
ATK3                 RQLQDRLVNSERRLQVSDL---STFEKMNEYEDQKQSIIDLKS-RVEEAELKLVEGEKLR
ATK5                 KMLEQELAFAKEKLKMVDL---SMSHTMTEFEEQKQCMHELQD-RLADTERQLFEGELLR
ATK1                 NMLEQQLAIANERQKMADA---SVSLTRTEFEEQKHLLCELQD-RLADMEHQLCEGELLR
Os03g64415           PPSTAAAGSCRRHPAAVTARWPLAGGARSGRRDNPRRQGTGAT-RTGISKTYVSSVVAKT
Os04g53760           QTLQKQLAVATNKLKLADV---TAIEAMTGYEEQKVIIKDLEE-RLASAEFQIVEADKLR
Os07g01490           KTLELQLASANEKLKRSDL---TTMETMTEYEKQKRMLEDLQL-RLEEAEQQILDGENLR
At2g22610            EIMKREQSQLSQEAHECVEGIPELYKMVGGVQALVS--------QCEDLKQKYSEEQAKR
Os03g02290           KAMKQEQTLLSLEAHDCANAVPDLSKMIGAVQTLVA--------QCEDLKLKYYEEMAKR
At1g72250            KIMKVEQIKLLEEATTYKHLVQDINEFSSHIQSRVKQDAELHE----NLKVKFVAGEKER
Os12g42160           KALKAEHQILLQESLECKKFVADATQMTTTIQQHVNQYASLEC-EFKDLKEKFTEETKER
At5g27550            KILKDEHALVSNQVQEIKN----CSLVEPEISRALQLLTT----KLGALEKQYLEESSER
Os05g33030           -------INTPDRLAALRFLGGKYNSLLERYKQQVAKCAEECAPRYDGLKKKYADECAER


Os06g11380           KRLFNDLLTLKGNVKVFCRSRPLFEDE-----GSSVVE-FPDDFTIRVNTGDESLTN-PK
AtKCA1               KRLFNDLLTAKGNIKVFCRARPLFEDE-----GPSVIE-FPGDCTICVNTSDDTLSN-PK
AtKCA2               KRLFNDLLTTKGNVKVFCRARPLFEDE-----GPSIIE-FPDNCTIRVNTSDDTLSN-PK
AtKinesin-12A        -----------SGVKVIVRMKPLNKG---------------EEGDMIVEKMSKDSLTVSG
At3g10310            RKLYNMVQDLKGNIRVYCRVRPIFNSEM----DGVIDY-IGKDGSLFVLDPSKPYKD-AR
Os01g14090           KSLYNLLQELRGNIRVFCRIRPLINSES----ISSIEH-IGNDGSIMVCDPLKPQT--TR
At1g63640            RRLYNEVQELKGNIRVYCRIRPFLPGQNS-R-QTTIEY-IGETGELVVANPFKQGKD-TH
At5g41310            RRLYNEVQELKGNIRVYCRIRPFLQGQNK-K-QTSIEY-TGENGELVVANPLKQGKD-TY
Os02g13580           RKLFNEIQELKGNIRVYCRIRPFRPGEDD-K-SSSVEY-IGDNGELVLSNPTKQGKE-GG
Os06g36080           RKLFNEVQELKGNIRVHCRIRPFLPGEDQ-T-STTIEY-VGDNGELILANPAKRGKE-GH
Os01g54080           QKLFNEVQELKGNIRVYCRVRPFLPGQDG-K-LTAIDY-IGENGEILIANPSKQGKE-GY
Os05g44560           QKLFNEVQELKGNIRVYCRVRPFLPGQDK-K-STTVDY-IGENGELLISNPFKQGKD-GH
At1g18410            QKLFNELQELKGNIRVYCRVRPFLRGQGA-S-KTVVEH-IGDHGELVVLNPTKPGKD-AH
At1g73860            RKLFNELQELKGNIRVFCRVRPFLPAQGA-A-NTVVEY-VGEDGELVVTNPTRPGKD-GL
Os11g44880           RKLYNQIQDLRGNIRVYCRVRPFLPGKVS---SSSSVA-GLEDRTITVMTPSKHGKD-AR
OsKCH1               RKLYNQIQDLKGNIRVYCRVRPFLPGHRS---LSSSVA-DTEERTITIITPTKYGKD-GC
Os03g18980           RKLYNQVQDLKGSIRVYCRVRPFLPGQV----SSCAVG-SIDEGNITIITPSKSGKE-GR
At2g47500            RKLYNQVQDLKGSIRVYCRVRPFLPGQSS---FSSTIG-NMEDDTIGINTASRHGK--SL
At1g09170            RKLYNQVQDLKGSIRVYCRVRPFLPGQKS-V-LTTVDH-L-EDSTLSIATPSKYGKE-GQ
ATK4                 RKLYNLVQDLKGNIRVYCRVRPFLPGQESGG-LSAVED-I-DEGTITIRVPSKYGKA-GQ
AtKP1                RLLYNEVQDLKGTIRVYCRVRPFFQEQKD-M-QSTVDY-IGENGNIIINNPFKQEKD-AR
OsKCH2               RKLYNQVQDLKGSIRVYCRVKPFLKMQTD-Q-RSTVDH-IGENGEIMIVNPQKQGKE-GR
AtKCBP               KRYYNTIEDMKGKIRVYCRIRPLNEKESS---EREKQM-LTTVDEFTVEHPWKDD---KR
Os04g57140           KRYYNTIEDMKGKIRVFCRLRPLNDKELI---EKDKNI-VCSPDEFTVAHPWKDDKS---
At1g55550            RQILNEFLDLKGNIRVFCRVKPLGATEKL---RPPVAS---DTRNVIIKLSET-----KR
Os01g15540           REALNNYLDLKGNIRVFCRIRPFHHEESY---SSRNLF-TLDESNVFLKVAET-----KR
At5g27950            KQVLNKIIDTKGSIRVFCRVRPFLLTERRPI-REPVSF-GPDNVVIRSAG--------SS
Os11g42800           QEALDKLIDLKGSIRVFCRVRPSISANNF---MTKSPV-TVENEKIVVRAVG------IK
ATK2                 KKLHNTIQELKGNIRVFCRVRPLLSGENSSE-EAKTIS-YPTSLEALGRGIDLLQNG-QS
ATK3                 KKLHNTILELKGNIRVFCRVRPLLPGENNGD-EGKTIS-YPTSLEALGRGIDLMQNA-QK
ATK5                 KKLHNTILELKGNIRVFCRVRPLLPDDGGRQ-EASVIA-YPTSTESLGRGIDVVQSG-NK
ATK1                 KKLHNTILELKGNIRVFCRVRPLLPDDGGRH-EATVIA-YPTSTEAQGRGVDLVQSG-NK
Os03g64415           LAL----QELKGNIRVFCRVRPLLPNE-----SGAVAY--PKSGENLGRGIELTHNG-QM
Os04g53760           KKLHNTILELKGNIRVFCRVRPLLQDNDSSGAEEALIS-YPTSVESAGRGIDLMNQG-QR
Os07g01490           KRLHNTILELKGNIRVFCRVRPLLPNE-----SGAVAY--PKSGENLGRGIELTHNA-QM
At2g22610            KELYNHIQETKGNIRVFCRCRPLNTEETS---TKSATI-VDFDGAKDGELGVITGNN-SK
Os03g02290           KKLHNIVEETKGNIRVFCRCRPLSKDETS---SGYKCA-VDFDGAKDGDIAIVNGGA-AK
At1g72250            KELYNKILELKGNIRVFCRCRPLNFEETE---AGVSMG-IDVESTKNGEVIVMSNGF-PK
Os12g42160           KDLYNKLIEVKGNIRVFCRCRPLNGEEIEEGASMAVDFESAKDGELIVRGHVS-----SK
At5g27550            KRLYNEVIELKGNIRVFCRCRPLNQAEIANG-CASVAE-FDTTQE--NELQILSSDS-SK
Os05g33030           RRLYNELIELRGNIRVFCRCRPLSTAEISNG-CSSIVQ-IDPSHE--TELQFVPSDK-DR
                                . :.*  * .*

Os06g11380           KDYEFDRVYGPHIGQGELFHDV-QPLVQSALDGYNVAIFAYGQSRSGKTHTLEGSSH---
AtKCA1               KDFEFDRVYGPHVGQAALFSDV-QPFVQSALDGSNVSILSYGQTNAGKTYTMEGSNH---
AtKCA2               KEFEFDRVYGPQVGQASLFSDV-QPFVQSALDGSNVSIFAYGQTHAGKTYTMEGSNQ---
AtKinesin-12A        QTFTFDSIANPESTQEQMFQLVGAPLVENCLSGFNSSVFAYGQTGSGKTYTMWGPANGLL
At3g10310            KTFQFNQVFGPTATQDDVFRET-QPLIRSVMDGYNVCIFAYGQTGSGKTYTMSGPPGR-S
Os01g14090           KIFQFNKIFGPTTTQDEVYKET-QYLIRSVMDGYNVCIFAYGQTGSGKTHTMCGPSGGLS
At1g63640            RLFKFNKVFDQAATQEEVFLDT-RPLIRSILDGYNVCIFAYGQTGSGKTYTMSGPSIT-S
```

```
At5g41310          RLFKFNKVFGPESTQEEVFLDT-RPMIRSILDGYNVCIFAYGQTGSGKTYTMSGPSIT-S
Os02g13580         KNFTFNKVFGPITTQDAVFKDI-QPLIRSVLDGYNVCIFAYGQTGSGKTYTMMGPEKA-T
Os06g36080         KLFKFNKVLGPSASQDEVFKEI-QPLIRSVLDGYNVCIFAYGQTGSGKTYTMTGPENA-T
Os01g54080         RMFKFNKVFGTHSSQAEVFSDI-QPLIRSVLDGFNVCIFAYGQTGSGKTYTMSGPGTS--
Os05g44560         RMFKFNKVFSPFSSQAEVFSDI-QPLIRSVLDGFNVCIFAYGQTGSGKTYTMSGPSTS--
At1g18410          RKFRFNKVYSPASTQAEVFSDI-KPLIRSVLDGYNVCIFAYGQTGSGKTYTMTGPDGA-S
At1g73860          RQFKFNKVYSPTASQADVFSDI-RPLVRSVLDGYNVCIFAYGQTGSGKTYTMTGPDGS-S
Os11g44880         KSFTFNRVFGPLATQEQVFADM-QPLIRSVLDGYNVCIFAYGQTGSGKTFTMSGPKVL-T
OsKCH1             KSFSFNRVFGPASTQEEVFSDM-QPLIRSVLDGFNVCIFAYGQTGSGKTFTMSGPKVL-T
Os03g18980         KTFSFNKVFGPSATQDEVFLDT-QPLIRSVLDGYNVCIFAYGQTGSGKTYTMSGPKNM-T
At2g47500          KSFTFNKVFGPSATQEEVFSDM-QPLIRSVLDGYNVCIFAYGQTGSGKTFTMSGPRDL-T
At1g09170          KTFTFNKVFGPSASQEAVFADT-QPLIRSVLDGYNVCIFAYGQTGSGKTFTMMGPNEL-T
ATK4               KPFMFNKVFGPSATQEEVFSDM-QPLVRSVLDGYNVCIFAYGQTGSGKTFTMTGPKEL-T
AtKP1              KIFSFNKVFGQTVSQEQIYIDT-QPVIRSVLDGFNVCIFAYGQTGSGKTYTMSGPDLM-T
OsKCH2             KMFSFNKIFGPNASQSEVFADT-QPLIRSVMDGYNVCIFAYGQTGSGKTYTMSGPDIT-T
AtKCBP             KQHIYDRVFDMRASQDDIFEDT-KYLVQSAVDGYNVCIFAYGQTGSGKTFTIYGHE----
Os04g57140         KQHIYDRVFDANTTQEEVFEDT-KYLVQSAVDGYNVCIFAYGQTGSGKTFTIYGSE----
At1g55550          KTYNFDRVFQPDSSQDDVFLEI-EPVIKSVIDGYNACIFAYGQTGTGKTYTMEGLP----
Os01g15540         KQYKFDKVFDQFSTQGDVFSEV-EPVIKSALDGYNVCIFAYGQTGSGKTYTMEGKP----
At5g27950          KEFEFDKVFHQSATQEEVFGEV-KPILRSALDGHNVCVLAYGQTGTGKTFTMDGTS----
Os11g42800         KEFSVDRVFDQESTQEDVFQEV-KPILRSALDGHNVCILAYGQTGTGKTYTMEGNN----
ATK2               HCFTFDKVFVPSASQEDVFVEI-SQLVQSALDGYKVCIFAYGQTGSGKTYTMMGRPGN--
ATK3               HAFTFDKVFAPTASQEDVFTEI-SQLVQSALDGYKVCIFAYGQTGSGKTYTMMGRPGN--
ATK5               HPFTFDKVFDHGASQEEVFFEI-SQLVQSALDGYKVCIFAYGQTGSGKTYTMMGRPET--
ATK1               HPFTFDKVFNHEASQEEVFFEI-SQLVQSALDGYKVCIFAYGQTGSGKTYTMMGRPEA--
Os03g64415         YFFTFDKVFEQSTSQEDVFIEI-SHLVQSALDGYKVCIFAYGQTGSGKTYTMMGNPEL--
Os04g53760         FSFSYDKVFDHGASQEDVFVEM-SQLVQSALDGYKVCIFAYGQTGSGKTYTMMGPPG---
Os07g01490         YSFTFDKVFEQSASQEDVFIEI-SQLIQSALDGYKVCIFAYGQTGSGKTYTMMGNPEL--
At2g22610          KSFKFDRVYTPKDGQVDVFADA-SPMVVSVLDGYNVCIFAYGQTGTGKTFTMEGTP----
Os03g02290         KTFKFDRVYMPTDNQADVYADA-SPLVTSVLDGYNVCIFAYGQTGTGKTFTMEGTE----
At1g72250          KSFKFDSVFGPNASQADVFEDT-APFATSVIDGYNVCIFAYGQTGTGKTFTMEGTQ----
Os12g42160         KVFKFDSVFSPEEDQEKVFEKT-VPFATSVLDGYNVCIFAYGQTGTGKTFTMEGIE----
At5g27550          KHFKFDHVFKPDDGQETVFAQT-KPIVTSVLDGYNVCIFAYGQTGTGKTFTMEGTP----
Os05g33030         KAFKFDHVFGPSDNQETVFAES-LPVVRSVMDGFNVCIFAYGQTGTGKTFTMEGIP----
                     .  : :      *  ::      .  . :.* : .:::***: :***.*: *

Os06g11380         -------DRGLYLRSFEELFDLSNSDT----TSTSHFNFYITACELYNDQVRDLLSDSI-
AtKCA1             -------DRGLYARCFEELFDLANSDS----TSTSRFSFSLSVFEIYNEQIRDLLSETQ-
AtKCA2             -------DRGLYARCFEELMDLANSDS----TSASQFSFSVSVFELYNEQVRDLLSGCQ-
AtKinesin-12A      EEHLCGDQRGLTPRVFERLFARIKEEQVKHAERQLNYQCRCSLLEIYNEQITDLLDPSQ-
At3g10310          AT-----EMGINYLALSDLFLICDR-R----KDMMTYEIYVQMVEIYNEQVRDLLAENS-
Os01g14090         SQ-----DLGISYMALNDLFKTSTS------REDVKYDIHVQMVEIYNEQVRDLLNEDT-
At1g63640          KE-----DWGVNYRALNDLFLLTQS-R----QNTVMYEVGVQMVEIYNEQVRDILSDGG-
At5g41310          EE-----DRGVNYRALNDLFHLTQS-R----QNSVMYEVGVQMVEIYNEQVRDLLSQD--
Os02g13580         EK-----EWGVNYRALNDLFNISHD-R----RDTITYELGVQMIEIYNEQIRDLLGSGG-
Os06g36080         EK-----DWGVNYRALNDLFHISRS-R----RDTVMYKVSVQMIEIYNEQIHDLLGNSG-
Os01g54080         RE-----DWGVNYRALNDLFDISLS-R----KNAFSYEVGVQMVEIYNEQVRDLLSNDI-
Os05g44560         KQ-----DWGVNYRALNDLFDISLS-R----RNAFSYEVGVQMVEIYNEQVRDLLSNDI-
At1g18410          EE-----EWGVNYRALNDLFRISQS-R----KSNIAYEVGVQMVEIYNEQVRDLLS----
At1g73860          EE-----DWGVNYRALNDLFKISQS-R----KGNISYEVGVQMVEIYNEQVLDLLSDDN-
Os11g44880         EE-----GLGVNYRALNDLFNIQAQ-R----KDTFCYEISVQMIEIYNEQVRDLLQNGG-
OsKCH1             EE-----SLGVNYRALNDLFNIKAQ-R----KGTIDYEISVQMIEIYNEQVRDLLQDGG-
Os03g18980         EQ-----TQGVNYRALSDLFKLAEQ-R----KGAFIYDIAVQMIEIYNEQVRDLLVNDG-
At2g47500          EK-----SQGVNYRALGDLFLLAEQ-R----KDTFRYDIAVQMIEIYNEQVRDLLVTDG-
At1g09170          DE-----TLGVNYRALSDLFHLSK------------------------------------
ATK4               EE-----SLGVNYRALADLFLLSNQ-R----KDTTSYEISVQMLEIYNEQVRDLLAQDG-
AtKP1              ET-----TWGVNYRALRDLFQLSNA-R----THVVTYEIGVQMIEIYNEQVRDLLVSDG-
OsKCH2             EE-----TWGVNYRSLNDLFAISQN-R----ADTTTYDVKVQMIEIYNEQVRDLLMVDG-
AtKCBP             -S-----NPGLTPRATKELFNILKR-D----SKRFSFSLKAYMVELYQDTLVDLLLPKS-
Os04g57140         -N-----NPGLTPRATSELFRVIKR-D----GHKYSFSLKAYMVELYQDNLVDLLLAKN-
At1g55550          -N-----SPGIVPRAIKGLFKQVEE-------SNHMFTIHFSMLEIYMGNLKDLLLSEA-
Os01g15540         -T-----NLGVIPRGIQTLFNQASE-------CNNRFLFTFSMLEIYMGNIRDLLAPRS-
At5g27950          -E-----QPGLAPRAIKELFNEASM------DQTHSVTFRMSMLEIYMGNLKDLLSARQ-
Os11g42800         -G-----KLGIVPRAIQELFSHASQ------DSSSTYSFSISMLEVYMGTVRDLLTPRQ-
ATK2               PD-----EKGLIPRCLEQIFQTRQSLR----SQGWKYELQVSMLEIYNETIRDLLSTNK-
ATK3               VE-----EKGLIPRCLEQIFETRQSLR----SQGWKYELQVSMLEIYNETIRDLLSTNK-
ATK5               PE-----QKGLIPRSLEQIFKTSQSLS----TQGWKYKMQVSMLEIYNESIRDLLSTSRT
ATK1               PD-----QKGLIPRSLEQIFQASQSLG----AQGWKYKMQVSMLEIYNETIRDLLSTNRT
Os03g64415         HD-----QKGLIPRSLEQIFQTSQALI----SQGWKYKMQASMLEIYNEAICDLLATNH-
Os04g53760         RD-----QKGIIPRSLEQIFKTSQSLE----SQGWKYSMQASMLEIYNETIRDLLAPGR-
Os07g01490         HD-----QKGLIPRSLEQIFQTSQALI----SQGWKYKMQASMLEIYNEAIRDLLATNR-
```

```
At2g22610          -Q-----NRGVNYRTVEQLFEVARE-R----RETISYNISVSVLEVYNEQIRDLLATSP-
Os03g02290         -R-----NRGVNYRTLEELFKIAEE-R----KETVTYSISVSVLEVYNEQIRDLLASSP-
At1g72250          -H-----DRGVNYRTLENLFRIIKA-R----EHRYNYEISVSVLEVYNEQIRDLLVPAS-
Os12g42160         -D-----ARGVNYRTLEELFRITKE-R----QGLFQYEITVSVLEVYNEQIHDLLLTGT-
At5g27550          -E-----NRGVNYRTLEELFRCSES-K----SHLMKFELSVSMLEVYNEKIRDLLVDNS-
Os05g33030         -E-----DRGVNYRALEELFRLSEE-R----SSSVAYTFAVSILEVYNEKIRDLLDESS-
                            *:       ::

Os06g11380         ---------SPVPKVRMGVQE--------SFVELVQEKVENPLEFSNSLKAALENRSANS
AtKCA1             ---------SNLPNINMDLHE--------SVIELGQEKVDNPLEFLGVLKSAFLNRGNYK
AtKCA2             ---------SNLPKINMGLRE--------SVIELSQEKVDNPSEFMRVLNSAFQNRGNDK
AtKinesin-12A      --------------KNLMIRE-DV-KSGVYVENLTEEYVKNLTDVSQLLIKGLGNRRTGA
At3g10310          ------------SCTRLDIRTCSSDDDGLSLPDATMHSVNSTKDVLQLMEAGEVNRAVSS
Os01g14090         ------------SSTKLDIRT-SS-NGLLNLPDAKKCPVQSPSDVINLMLLGEKHRASSP
At1g63640          ------------SSRRLGIWN-TALPNGLAVPDASMHCVRSTEDVLELMNIGLMNRTVGA
At5g41310          -------------------------------VPDASMHSVRSTEDVLELMNIGLMNRTVGA
Os02g13580         ------------VQKKLGIQN-TIQPNGLAVPDATMCPVTSTSHVIELMQTGHDNRAMSA
Os06g36080         ------------SEKKLGILN-ASQPNGLAVPDATMHPVNSSSDVIELMRTGLENRSVGA
Os01g54080         ------------AQKRLGIWS-TSQPNGLVVPDASLHPVKSTSDVLDLMEIGQSNRAVGS
Os05g44560         ------------AQKRLGIWS-TSQPNGLVVPDASLHPVKSTSDVLDLMEIGQANRAVGS
At1g18410          -----------------GILS-TTQQNGLAVPDASMYPVTSTSDVLELMSIGLQNRVVSS
At1g73860          -----------SQKKTLGILS-TTQQNGLAVPDASMYPVTSTSDVITLMDIGLQNRAVGS
Os11g44880         -------------NKKVDIKN-SS-QKGIAVPDANIVPVTSTSDVIDLMNLGQKNRAVCS
OsKCH1             -------------NRRLEIRN-TP-QKGLAVPDASIVPVTSTADVVELMNQGQKNRAVGS
Os03g18980         ------------LNKRLEIRN-NS-QNGLNVPDASLVCVASTMDVMELMNVGQKNRAVGA
At2g47500          ------------SNKRLEIRN-SS-QKGLSVPDASLVPVSSTFDVIDLMKTGHKNRAVGS
At1g09170          ------------------IRN-ST-QDGINVPEATLVPVSTTSDVIHLMNIGQKNRAVSA
ATK4               ------------QTKRLEIRN-NS-HNGINVPEASLVPVSSTDDVIQLMDLGHMNRAVSS
AtKP1              ------------SSRRLDIRN-NSQLNGLNVPDANLIPVSNTRDVLDLMRIGQKNRAVGA
OsKCH2             ------------ANKRLEIRN-SSHVNGLNIPDANLVPVKCAQDVLDLMRVGHRNRAVGS
AtKCBP             -----------ARRLKLEIKK-DS-KGMVFVENVTTIPISTLEELRMILERGSERRHVSG
Os04g57140         -----------ATHQKLEIKK-DS-KGVVTVENVTVVNISSFEELRAIILRGSERRHTAG
At1g55550          ------TKPISPIPPSLSIHT-DP-NGEIDIENLVKLKVDDFNEILRLYKVGCRSRATAS
Os01g15540         ------KTNGIKNVPSLSIKS-DP-DGGIEIEDLVAVTVNSFQEVKRLYEMGTRLRSTAS
At5g27950          ----SLKSYEASAKCNLNIQV-DS-KGSVEIEGLTEVEVMDFTKARWWYNKGRRVRSTSW
Os11g42800         ---PLFRSTECNTSSIISILA-TK-SGAVEVEGLTDVAIQDLKKANQWYCRGRRARSTSW
ATK2               ---EAVRADNGVSPQKYAIKH-DA-SGNTHVVELTVVDVRSSKQVSFLLDHAARNRSVGK
ATK3               ---EAVRTDSGVSPQKHAIKH-DA-SGNTHVAELTILDVKSSREVSFLLDHAARNRSVGK
ATK5               IAIESVRADSSTSGRQYTITH-DV-NGNTHVSDLTIVDVCSIGQISSLLQQAAQSRSVGK
ATK1               TSMDLVRADSGTSGKQYTITH-DV-NGHTHVSDLTIFDVCSVGKISSLLQQAAQSRSVGK
Os03g64415         ------TTIQDGGASKYSIKH-DA-NGNTHVSDLIIVDVLSINEVSSLLKRAAQSRSVGR
Os04g53760         -----SNNFDLSTSKQYTIKH-DP-QGNTTVTDLTVADVFSSADVTSLLAKASQSRSVGR
Os07g01490         ------TTVQDGGASKYSIKH-DA-NGNTHVSDLTIVDVSSINEVSSLLKRAAQSRSVGR
At2g22610          ------------GSKKLEIKQ-SS-DGSHHVPGLVEANVENINEVWNVLQAGSNARSVGS
Os03g02290         ------------SSKKLEIKQ-AS-EGSHHVPGIVEAKVENIKEVWDVLQAGSNARAVGS
At1g72250          --------QSASAPKRFEIRQ-LS-EGNHHVPGLVEAPVKSIEEVWDVLKTGSNARAVGK
Os12g42160         --------QPGATAKRLEVRQ-VA-EGVHHVPGLVEARVTNMNEAWEVLQTGSKARVVGS
At5g27550          ----------NQPPKKLEVKQ-SA-EGTQEVPGLVEAQVYNTDGVWDLLKKGYAVRSVGS
Os05g33030         ----------EQTGRKLDIKQ-TA-DGTQEVAGLIEAPIYTIDGVWEKLKVGAKNRSVGA
                                                 .       :            .   *

Os06g11380         LKVMVS----HLIVTIHI--HYRNYVTG-EHLY-SKLSLVDLPASECLLEEDANRDNVTD
AtKCA1             SKFNVT----HLIVSIHI--YYSNTITG-ENIY-SKLSLVDLAGSEGLIMENDSGDHVTD
AtKCA2             S----KSTVTHLIVSIHI--CYSNTITR-ENVI-SKLSLVDLAGSEGLTVEDDNGDHVTD
AtKinesin-12A      TSVNTESSRSHCVFTCVVESRCKNVADGLSSFKTSRINLVDLAGSERQKSTGAAGERLKE
At3g10310          TSMNNRSSRSHSIFMVHV--RGKDT-SG-GTLR-SCLHLVDLAGSERVDKSEVTGDRLKE
Os01g14090         TAMNHRSSRSHSILTVHV--NGKDM-SG-NVTR-SSLHLVDLAGSERVDRSEATGDRLKE
At1g63640          TALNERSSRSHCVLSVHV--RGVDVETD-SILR-GSLHLVDLAGSERVDRSEATGERLKE
At5g41310          TTLNEKSSRSHSVLSVHV--RGVDVKTE-SVLR-GSLHLVDLAGSERVGRSEVTGERLKE
Os02g13580         TALNERSSRSHSVVTIHV--RGQDLKTG-NTLR-GALHLVDLAGSERVDRSAVTGDRLKE
Os06g36080         TALNERSSRSHSVVTMHI--QGVDLKTG-VTLR-GALHLVDLAGSERVDRSAATGDRLKE
Os01g54080         TALNERSSRSHSILTVHV--RGLDVKNG-STSR-GCLHLIDLAGSERVERSEATGDRLKE
Os05g44560         TALNERSSRSHSILTVHV--RGLDVKNG-STSR-GCLHLIDLAGSERVERSEATGDRLKE
At1g18410          TALNERSSRSHSIVTVHV--RGKDLKTG-SALY-GNLHLVDLAGSERVDRSEVTGDRLKE
At1g73860          TALNERSSRSHSIVTVHV--RGKDLKTG-SVLY-GNLHLVDLAGSERVDRSEVTGDRLRE
Os11g44880         TAMNDRSSRSHSCLTVHV--QGRDLTSR-TVLR-GCMHLVDLAGSERVDKSEVVGDRLKE
OsKCH1             TAINDRSSRSHSCLSVHV--QGKYLTSG-AMLR-GCMHLVDLAGSERVDKSEVVGDRLKE
Os03g18980         TALNDRSSRSHSCLTVHV--QGRDLTSG-TILR-GCMHLVDLAGSERVDKSEVTGERLKE
At2g47500          TALNDRSSRSHSCLTVHV--QGRDLTSG-AVLR-GCMHLVDLAGSERVDKSEVTGDRLKE
At1g09170          TAMNDRSSRSHSCLTVHV--QGKDLTSG-VTLR-GSMHLVDLAGSERIDKSEVTGDRLKE
ATK4               TAMNDRSSRSHSCVTVHV--QGRDLTSG-SILH-GSMHLVDLAGSERVDKSEVTGDRLKE
```

```
AtKP1                 TALNERSSRSHSVLTVHV--QGKELASG-SILR-GCLHLVDLAGSERVEKSEAVGERLKE
OsKCH2                TALNERSSRSHSVLTVHV--QGKEIASG-STLR-GCLHLVDLAGSERVDKSEAAGERLNE
AtKCBP                TNMNEESSRSHLILSVVI--ESIDLQTQ-SAAR-GKLSFVDLAGSERVKKSGSAGCQLKE
Os04g57140            TNMNVESSRSHLILSIII--ESTNLQTQ-SYAR-GKLSFVDLAGSERVKKSGSAGKQLKE
At1g55550             TNSNSVSSRSHCMIRVSV--TSLGAPER-RRET-NKIWLVDLGGSERVLKTRATGRRFDE
Os01g15540            TMANSTSSRSHCLIRISL--TSLNATER-RKAT-SKLWMIDLGGSERLVKTKATGKRLKE
At5g27950             TNVNETSSRSHCLTRITI--FRRGDAVG-SKTEVSKLWMIDLGGSERLLKTGAIGQTMDE
Os11g42800            TNVNDVSSRSHCLTRITI----KRSSGG-TTEECSKLWLVDLGGSERLLKTGASGLTMDE
ATK2                  TAMNEQSSRSHFVFTLKI--SGFNESTE-QQVQ-GVLNLIDLAGSERLSKSGSTGDRLKE
ATK3                  TQMNEQSSRSHFVFTLRI--SGVNESTE-QQVQ-GVLNLIDLAGSERLSKSGSTGDRLKE
ATK5                  THMNEQSSRSHFVFTLRI--SGVNESTE-QQVQ-GVLNLIDLAGSERLSRSGATGDRLKE
ATK1                  TQMNEQSSRSHFVFTMRI--SGVNESTE-QQVQ-GVLNLIDLAGSERLSKSGATGDRLKE
Os03g64415            TQMNEESSRSHCVFTLRF--FGVNEGTD-QQVQ-GVLNLIDLAGSERLNKSGATGDRLKE
Os04g53760            TQMNEQSSRSHFVFTLKI--SGSNENTG-QQVQ-GVLNLIDLAGSERLAKSGSTGDRLKE
Os07g01490            TQMNEESSRSHCVFTLRI--FGVNEGTD-QQVQ-GVLNLIDLAGSERLNKSGATGDRLKE
At2g22610             NNVNEHSSRSHCMLSIMV--KAKNLMNG-DCTK-SKLWLVDLAGSERLAKTDVQGERLKE
Os03g02290            NNVNEHSSRSHCMLCIMV--RAENLMNG-ECTR-SKLWLVDLAGSERLAKTDVQGERLKE
At1g72250             TTANEHSSRSHCIHCVMV--KGENLLNG-ECTK-SKLWLVDLAGSERVAKTEVQGERLKE
Os12g42160            TNANEHSSRSHCMHCVMV--KGENLMNG-EQTK-SKLWLIDLAGSERVAKTDAQGERLKE
At5g27550             TAANEQSSRSHCLLRVTV--KGENLING-QRTR-SHLWLVDLAGSERVGKVEVEGERLKE
Os05g33030            TSANELSSRSHSLVKVTV--RSEHLVTG-QKWR-SHIWLVDLAGSERVNKTEVEGDRLKE
                                *      .                . : ::** .**           . :

Os06g11380            FLHVSKSLSALGDALASLSA-----KKEPVLSGNSRITQILADSLGSSSKTLLIVHVSPS
AtKCA1                LLHVMNSISALGDVLSSLTS-----GKDSIPYDNSILTRVLADSLGGSSKTLMIVNICPS
AtKCA2                LLHVTNSISALGDVLSSLTS-----KRDTIPYENSFLTRILADSLGGSSKTLMIVNICPS
AtKinesin-12A         AGNINRSLSQLGNLINILAEISQTGKPRHIPYRDSRLTFLLQESLGGNAKLAMVCAVSPS
At3g10310             AQYINKSLSCLGDVISALAQ-----KNSHIPYRNSKLTLLLQDSLGGQAKTLMFAHLSPE
Os01g14090            AQHINKSLSCLGDVITALAQ-----KNSHIPYRNSKLTQLLQSSLGGNAKTLMFAHISPE
At1g63640             AQHINKSLSALGDVIFALAH-----KNPHVPYRNSKLTQVLQSSLGGQAKTLMFVQVNPD
At5g41310             AQHINKSLSALGDVIFALAH-----KNPHVPYRNSKLTQVLQNSLGGQAKTLMFVQINPD
Os02g13580            AQHINKSLAALGDVIFSLSQ-----KNAHVPYRNSKLTQVLQTSLGGHAKTLMFVQVNPD
Os06g36080            AQHINKSLSALGDVIFSLSQ-----KNAHVPYRNSKLTQVLQNSLGGNAKTLMFVQVNPD
Os01g54080            AQHINKSLSALGDVIFSLAQ-----KNAHVPYRNSKLTQVLQSSLGGQAKTLMLVQINPD
Os05g44560            AQHINKSLSALGDVIFALAQ-----KNAHVPYRNSKLTQVLQSSLGGQAKTLMFVQINPD
At1g18410             AQHINKSLSALGDVIFSLAS-----KSSHVPYRNSKLTQLLQSSLGGRAKTLMFVQLNPD
At1g73860             AQHINKSLSSLGDVIFSLAS-----KSSHVPYRNSKLTQLLQTSLGGRAKTLMFVQLNPD
Os11g44880            AQHINKSLAALGDVIASLAQ-----KNAHVPYRNSKLTQLLQDSLGGQAKTLMFVHIAPE
OsKCH1                AQYINKSLSALGDVIASLAQ-----KNSHVPYRNSKLTQLLQDSLGGQAKTLMFVHVSPE
Os03g18980            AQHINKSLSALGDVIASLAQ-----KSAHVPYRNSKLTQLLQDSLGGQAKTLMFVHISPE
At2g47500             AQHINRSLSALGDVIASLAH-----KNPHVPYRNSKLTQLLQDSLGGQAKTLMFVHISPE
At1g09170             AQHINKSLSALGDVIASLSQ-----KNNHIPYRNSKLTQLLQDALGGQAKTLMFIHISPE
ATK4                  AQHINKSLSALGDVISSLSQ-----KTSHVPYRNSKLTQLLQDSLGGSAKTLMFVHISPE
AtKP1                 AQHINKSLSALGDVIYALAQ-----KSSHVPYRNSKLTQVLQDSLGGQAKTLMFVHINPE
OsKCH2                AKHINKSLSALGDVIAALAQ-----KSSHVPYRNSKLTQVLQDALGGQAKTLMFVHMNPE
AtKCBP                AQSINKSLSALGDVIGALSS-----GNQHIPYRNHKLTMLMSDSLGGNAKTLMFVNVSPA
Os04g57140            AQSINKSLSALADVIGALSS-----DGQHIPYRNHKLTMLMSDSLGGNAKTLMFVNVSPA
At1g55550             GKAINLSLSALGDVINSLQR-----KNSHIPYRNSKLTQVLKDSLGQDSKTLMLVHISPK
Os01g15540            GKAINLSLSALGDVIDALQT-----KKPHVPYRNSKLTQVLRDSLGCESKTLMLVHISPD
At5g27950             GRAINLSLSALGDVIAALRR-----KKGHVPYRNSKLTQILKDSLGTRSKVLMLVHISPR
Os11g42800            GKAINLSLSALGDVIAALRR-----KRSHVPYRNSKLTQILSDSLGDGSKVLMVVHISPS
ATK2                  TQAINKSLSSLGDVIFALAK-----KEDHVPFRNSKLTYLLQPCLGGDSKTLMFVNITPE
ATK3                  TQAINKSLSSLGDVIFALAK-----KEDHVPFRNSKLTYLLQPCLGGDAKTLMFVNIAPE
ATK5                  TQAINKSLSALSDVIFALAK-----KEDHVPFRNSKLTYLLQPCLGGDSKTLMFVNISPD
ATK1                  TQAINKSLSALSDVIFALAK-----KEDHVPFRNSKLTYLLQPCLGGDSKTLMFVNISPD
Os03g64415            TQAINKSLSCLSDVIFSIAK-----KEEHVPFRNSKLTYLLQPCLGGDSKTLMFVNLSPE
Os04g53760            TQAINKSLSALSDVIFAIAK-----GDDHVPFRNSKLTYLLQPCLGGDSKTLMFVNISPE
Os07g01490            TQAINKSLSCLSDVIFSIAK-----KEEHVPFRNSKLTYLLQPCLGGDSKTLMFVNLSPE
At2g22610             AQNINRSLSALGDVIYALAT-----KSSHIPYRNSKLTHLLQDSLGGDSKTLMFVQISPS
Os03g02290            AQNINRSLSALGDVISALAT-----KNSHIPYRNSKLTHLLQDSLGGDSKALMFVQISPS
At1g72250             TQNINKSLSALGDVIFALAN-----KSSHIPFRNSKLTHLLQDSLGGDSKTLMFVQISPN
Os12g42160            AQNINKSLSALGDVISALAT-----KSQHIPFRNSKLTHLLQDSLSGDSKTLMFVQISPN
At5g27550             SQFINKSLSALGDVISALAS-----KTSHIPYRNSKLTHMLQNSLGGDCKTLMFVQISPS
Os05g33030            SQFINKSLSALGDVISALAS-----KNAHIPYRNSKLTHLLQSSLGGDCKTLMFVQISPS
                         :  *:: *.: :  :           :   :  :* ::  .*.  .*  :.  : *

Os06g11380            ASNLSRTLSTLSFSARAKNAE---------------------------------------
AtKCA1                VQTLSETISCLNYAARARNTV---------------------------------------
AtKCA2                ARNLSEIMSCLNYAARARNTV---------------------------------------
AtKinesin-12A         QSCRSETFSTLRFAQRAKAIQNKAVVNEVMQDDVNFLRGVIHQLRDELQRMKNDGNNPTN
At3g10310             EDSFGETISTLKFAQRVSTVE---------------------------------------
```

```
Os01g14090          ADSYVETLSTLKFAQRASCVE---------------------------------------
At1g63640           GDSYAETVSTLKFAERVSGVE---------------------------------------
At5g41310           EDSYAETVSTLKFAERVSGVE---------------------------------------
Os02g13580          VSSYTETLSTLKFAERVSGVE---------------------------------------
Os06g36080          VSSYAETLSTLKFADRVSGVE---------------------------------------
Os01g54080          IESYSETISTLKLAERVSGVE---------------------------------------
Os05g44560          VESYSETISTLKFAERVSGVE---------------------------------------
At1g18410           ITSYSESMSTLKFAERVSGVE---------------------------------------
At1g73860           ATSYSESMSTLKFAERVSGVE---------------------------------------
Os11g44880          PDAIGESISTLKFAERVATVE---------------------------------------
OsKCH1              LDAVGETISTLKFAERVASVE---------------------------------------
Os03g18980          SDALGESISTLKFAERVSTVE---------------------------------------
At2g47500           ADAVGETISTLKFAERVATVE---------------------------------------
At1g09170           LEDLGETLSTLKFAERVATVD---------------------------------------
ATK4                PDTLGETISTLKFAERVGSVE---------------------------------------
AtKP1               VNAVGETISTLKFAQRVASIE---------------------------------------
OsKCH2              ADAFGETMSTLKFAERVATVE---------------------------------------
AtKCBP              ESNLDETYNSLLYASRVRTIV---------------------------------------
Os04g57140          ESNLEETYNSLMYASRVRCIV---------------------------------------
At1g55550           EDDLCETICSLNFATRAKNIH---------------------------------------
Os01g15540          EGDLCETICTLGFATRVRSIR---------------------------------------
At5g27950           DEDVGETICSLSFTKRARAVE---------------------------------------
Os11g42800          DDDIGETVCSLSFAKRARSIE---------------------------------------
ATK2                PSSTGESLCSLRFAARVNACE---------------------------------------
ATK3                SSSTGESLCSLRFAARVNACE---------------------------------------
ATK5                PSSTGESLCSLRFAARVNACE---------------------------------------
ATK1                PTSAGESLCSLRFAARVNACE---------------------------------------
Os03g64415          VSSTGESICSLRFAARVNSCE---------------------------------------
Os04g53760          ASSVGETICSLRFASRVNACE---------------------------------------
Os07g01490          VSSTGESICSLRFAARVNSCE---------------------------------------
At2g22610           EHDVSETLSSLNFATRVRGVE---------------------------------------
Os03g02290          NNDVSETLSSLNFASRVRRIE---------------------------------------
At1g72250           ENDQSETLCSLNFASRVRGIE---------------------------------------
Os12g42160          ENDVGETLCSLNFASRVRGIE---------------------------------------
At5g27550           SADLGETLCSLNFASRVRGIE---------------------------------------
Os05g33030          SADSGETLCSLNFASRVRAID---------------------------------------
                              *  : *.

Os06g11380          ----------------------LS----LGNRDTIKKWKDVANDSRKELHDKEKEVLDLR
AtKCA1              ----------------------PS----LGNRDTIKKWRDVASDARKELLEKERENQNLK
AtKCA2              ----------------------PS----LGNRDTIKKWRDVANDARKEVLEKERENQRLK
AtKinesin-12A       PNVAYSTAWNARRSLNLLRSFGLGHPRSLPHED---N--------------DGDIEMEID
At3g10310           ----------------------LG----AARAH---K--------------ETREVMHLK
Os01g14090          ----------------------LG----TAHAN---K--------------ESNEIRELK
At1g63640           ----------------------LG----AAKSS---K--------------EGRDVRQLM
At5g41310           ----------------------LG----AARSY---K--------------EGRDVRQLM
Os02g13580          ----------------------LG----VARSNKEGK--------------EGKDVKELM
Os06g36080          ----------------------LG----AAKAN---K--------------EGKDIKEFK
Os01g54080          ----------------------LG----AARSN---R--------------EGKDIKELL
Os05g44560          ----------------------LG----AARSN---K--------------EGKDIKELL
At1g18410           ----------------------LG----AAKSS---K--------------DGRDVRELM
At1g73860           ----------------------LG----AAKTS---K--------------EGKDVRDLM
Os11g44880          ----------------------LG----AAKSN---K--------------EGGEVKELK
OsKCH1              ----------------------LG----AAKAN---K--------------EGSEVRELK
Os03g18980          ----------------------LG----AARLN---K--------------ESGEVKELK
At2g47500           ----------------------LG----AARVN---N--------------DTSDVKELK
At1g09170           ----------------------LG----AARVN---K--------------DTSEVKELK
ATK4                ----------------------LG----AARVN---K--------------DNSEVKELK
AtKP1               ----------------------LG----AARSN---K--------------ETGEIRDLK
OsKCH2              ----------------------LG----AAHAN---K--------------EVGQVKDLK
AtKCBP              ----------------------ND----PSKHI------------------SSKEMVRLK
Os04g57140          ----------------------ND----TSKHV------------------APKEIMRLK
At1g55550           ----------------------LG----QDEST------------------EEQAKKEAV
Os01g15540          ----------------------LESEEPPEMKA---RKETLLIDLGQKVNDLEHECEDIR
At5g27950           ----------------------------SNRGL-------------------TAELQKLR
Os11g42800          ----------------------------SSKEL-------------------SEDIKKLK
ATK2                ----------------------IG----TAHRH--------------------VNARPLD
ATK3                ----------------------IG----TPRRQ--------------------TNIKPLE
ATK5                ----------------------IG----IPRRQ--------------------TSAKLLD
ATK1                ----------------------IG----IPRRQ--------------------TSTKLLD
Os03g64415          ----------------------IG----IPRRQ--------------------TQVRSLA
```

```
Os04g53760        ----------------------IG----IPRRH--------------------TQARSFD
Os07g01490        ----------------------IG----IPRRQ--------------------TQVRSLA
At2g22610         ----------------------LG----PARKQ---V--------------DTGEIQKLK
Os03g02290        ----------------------LG----PAKKQ---V--------------DTAELQKVK
At1g72250         ----------------------LG----PAKKQ---L--------------DNTELLKYK
Os12g42160        ----------------------LG----QARKQ---V--------------DVGELSRYK
At5g27550         ----------------------SG----PARKQ--------------------ADVSELL
Os05g33030        ----------------------HG----PARKQ---A--------------DPAETFKLK


Os06g11380        QE-VLGLKLSLKEANDQCTLLFNEVQKAWRVSSTLQADLKSENLMLAEKHRIEKEQNNQL
AtKCA1            QE-VVGLKKALKDANDQCVLLYSEVQRAWKVSFTLQSDLKSENIMLVDKHRLEKEQNSQL
AtKCA2            QE-VTGLKQALKEANDQCVLLYNEVQRAWRVSFTLQSDLKSENAMVVDKHKIEKEQNFQL
AtKinesin-12A     EAAVERLCVQVGLQSSLASEGINHDMNRVKSIHSSDGQSIEKRLPEDSDVAMEDACCHTE
At3g10310         EQ-IENLKRALGTE----------------------------------------------
Os01g14090        EQ-VENLKRALAAKEL--------------------------------------------
At1g63640         EQ-VSNLKDVIAKKDE--------------------------------------------
At5g41310         EQ-VSNLKDMIAKKDE--------------------------------------------
Os02g13580        DQ-LSLLKDTISKKDE--------------------------------------------
Os06g36080        EQ-LSLLKDKIAKKDE--------------------------------------------
Os01g54080        EQ-VASLKDTIARKDM--------------------------------------------
Os05g44560        EQ-VASLKDTIVRKDT--------------------------------------------
At1g18410         EQ-LGSLKDTIARKDD--------------------------------------------
At1g73860         EQ-LASLKDTIARKDE--------------------------------------------
Os11g44880        EQ-IACLKAALAKKDG--------------------------------------------
OsKCH1            EQ-IATLKAALAKKEG--------------------------------------------
Os03g18980        EQ-IARLKSSLAMKDS--------------------------------------------
At2g47500         EQ-IATLKAALARKEA--------------------------------------------
At1g09170         EQ-IASLKLALARKES--------------------------------------------
ATK4              EQ-IANLKMALVRKGN--------------------------------------------
AtKP1             DE-ISSLKSAMEKKEA--------------------------------------------
OsKCH2            EE-ISKLKLALDDKER--------------------------------------------
AtKCBP            KL-VAYWKEQAGKKGE--------------------------------------------
Os04g57140        KL-IAYWKEQAGKRSE--------------------------------------------
At1g55550         MMNLQKMMEKIEQERE--------------------------------------------
Os01g15540        RK-IKNLEESMEHLTG-------------------------------PQPTIYSNFDMSH
At5g27950         EKKISELEEEMEETQE--------------------------------------------
Os11g42800        QKRIAELDKEICDAEQ--------------------------------------------
ATK2              YR-LSLG-----------------------------------------------------
ATK3              NR-LSLG-----------------------------------------------------
ATK5              SR-LSYG-----------------------------------------------------
ATK1              SR-LSYG-----------------------------------------------------
Os03g64415        QG----------------------------------------------------------
Os04g53760        SR-LSYG-----------------------------------------------------
Os07g01490        QG----------------------------------------------------------
At2g22610         AM-VEKARQESRSKDE--------------------------------------------
Os03g02290        QM-LERAKQDIRLKDD--------------------------------------------
At1g72250         QM-VEKWKQDMKGKDE--------------------------------------------
Os12g42160        LM-AGRAKQDSKNKDA--------------------------------------------
At5g27550         KS--KQMAEKLKHEEK--------------------------------------------
Os05g33030        QM-TEKIRHEEKENAK------------------------------------LLESLQLT


Os06g11380        RDQISRLLEVEQEQKIKMHERDLTIQSLQAKLKSIESQLNEALNSSDARSTIGSESASVI
AtKCA1            RNQIAQFLQLDQEQKLQMQQQDSAIQNLQAKITDLESQVSEAVRSDTTRTGDALQSQDIF
AtKCA2            RNQIAQLLQLEQEQKLQAQQQDSTIQNLQSKVKDLESQLSKALKSDMTRSRDPLE-----
AtKinesin-12A     NHEPETVDNMRTETETGIRENQIKTHSQTLDHESSFQPLSVKD-----------------
At3g10310         --EWNNVSNGSKEIKSPFSRPIATTERTPPRLRRLSIENCSST-----------------
Os01g14090        --EKSSFKLKENTVVRERAKQVPERTPPRPRRLSLENTGIGKG-----------------
At1g63640         --ELQNFQKVKGNNATSLKRGLSNLRLVGPTSPRRHSIGASPN-----------------
At5g41310         --ELQKFQNINGIQKRGLSKLRIVSPPRRHSLGGALTNSPRRR-----------------
Os02g13580        --EIDRLQLLNSSTRLKPTRQADSVLKHSSSSPGITSLGKGTS-----------------
Os06g36080        --EISRLQLQSHNTPRATAKRADSLLKHSSSSPGISSLGSKIQ-----------------
Os01g54080        --EIEQLQLLKSKSPNSMTDRNGSNLLRQSTSSTGLSSLPVAS-----------------
Os05g44560        --EIEQLQLMKDKVKSPSFAVDINGASMPKNSNSDLRSVLSIT-----------------
At1g18410         --EIERLHLLKDINYPQRLQKKSLGQSDDFNSEAGDSQLSIED-----------------
At1g73860         --EIERLQHQPQRLQKSMMRRKSIGHTDDINSDTGEY-----------------------
Os11g44880        --ETESIRSTQSSPDIYRMRMGSAPPAFRNPMEEVGNLETRSN-----------------
OsKCH1            --EPENIQSTQSSPDMYRIKRGNAIPAFPKNRQPMEEVGNLEV-----------------
Os03g18980        --GSEQNINRDPEAFNMKMPSPGFSNRRQGSCELVSSQTNF-------------------
At2g47500         -----------ESQQNNILKTPGGSEKHKAKTGEVEIHNNNIM-----------------
```

```
At1g09170           --GADQTQLQRPLTPDKLLRKKSLGVSSSFSKSANSTRQVQTK-----------------
ATK4                -GNDVQPTAIPINRERISRRRSLETPTIRPKLPTMGNTSNNSR-----------------
AtKP1               --ELEQLRSGSIRNTTECQRARAVSPFHLPRTGNGAGTKAEAS-----------------
OsKCH2              --EASKLRDIANRVASEKRNARTRSPLTTTLSSKPEAGQDSSV-----------------
AtKCBP              -------EEDLVDIEEDRTRKDEADS----------------------------------
Os04g57140          -------DDDLEEIQEERTPKEKADNRLTS------------------------------
At1g55550           -MSLRKMRNLNETLEKLTGKPHVIEEEEKDVVREVIHVTPKKP-----------------
Os01g15540          LSSEELKTDVSSNVRNSKNRREASSRLPRFMKPTASSQHRIGL-----------------
At5g27950           --GCKKIKARLQEVECLVNEHKKLFWITNDKYLEDIEKKAISP-----------------
Os11g42800          --ELKDLNEQIKRAETSLEERKKLSSSACQALSDEKGSPRSTL-----------------
ATK2                ------------------------------------------------------------
ATK3                ------------------------------------------------------------
ATK5                ------------------------------------------------------------
ATK1                ------------------------------------------------------------
Os03g64415          ------------------------------------------------------------
Os04g53760          ------------------------------------------------------------
Os07g01490          ------------------------------------------------------------
At2g22610           --SIKKMEENIQNLEGKNKGRDNSYRSLQEKNKDLQNQLDSVH-----------------
Os03g02290          --SLRKLEDNCQNLENKAKGKEQFYKNLQEKVKELESQLDSKM-----------------
At1g72250           --QIRKMEETMYGLEAKIKERDTKNKTLQDKVKELESQLLVER-----------------
Os12g42160          --QIKSMEETIQSLEAKNKAKDLLTMNLQEKIKELEAQLLVER-----------------
At5g27550           --ETKKLQDNVQSLQLRLTAREHICRGLQDKVRDLEFQLAEER-----------------
Os05g33030          QLKYASRENVIKTLQEKIREAEQTSKTYQQRVRELENELANEK-----------------


Os06g11380          -STPKMMESTADSSSVTKRLEEELAKRDALIEKLHEENEKLFDRLTEKSGLGSSPQAPSP
AtKCA1              SPIPKAVEGTTDSSSVTKKLEEELKKRDALIERLHEENEKLFDRLTERS-MAVSTQVLSP
AtKCA2              -PQPRAAENTLDSSAVTKKLEEELKKRDALIERLHEENEKLFDRLTEKS-VASSTQVSSP
AtKinesin-12A       -ALCSSLNKSEDVSSCPDLVPQDVTSANVLIADGVDDPEHLVNSASPSLCID--------
At3g10310           -KANLEDRRGIKS-----------------------------------------------
Os01g14090          -SIPDRKGPKSPL-----------------------------------------------
At1g63640           -ARRGKASGLF-------------------------------------------------
At5g41310           -QGPGLLGRTTSD-----------------------------------------------
Os02g13580          ------------------------------------------------------------
Os06g36080          -HRRTASGGRIKI-----------------------------------------------
Os01g54080          -QQNQQLSGSVEA-----------------------------------------------
Os05g44560          -TNQQSQLSDPQS-----------------------------------------------
At1g18410           -DSRFQHDYTRQS-----------------------------------------------
At1g73860           ------------------------------------------------------------
Os11g44880          -GTPRQKKRNFELPDVEN------------------------------------------
OsKCH1              -RNNATPMQKKASFQFSGVLSEN-------------------------------------
Os03g18980          -RQPMEDVGNIEVRANPTLRQ---------------------------------------
At2g47500           -TKKSESCEVEEI-----------------------------------------------
At1g09170           -HKPSQIDDVNSIEG---------------------------------------------
ATK4                -PQIMDLSGPEAFNDST-------------------------------------------
AtKP1               -PQPNDGTRSYETRSCSTGKQRKSGFPSALRNRE--------------------------
OsKCH2              -DTCTSEIRSSS------------------------------------------------
AtKCBP              ------------------------------------------------------------
Os04g57140          ------------------------------------------------------------
At1g55550           -RNKSRRASDVFPSFMRPTASSNRRLSGADFSVTPN------------------------
Os01g15540          -NNRTPIINRLKPPVPPRRRPSSVYAESVMVPVNAAP-----------------------
At5g27950           -LDHLKDTDATPI-----------------------------------------------
Os11g42800          -VVVGHIDSAESP-----------------------------------------------
ATK2                ------------------------------------------------------------
ATK3                ------------------------------------------------------------
ATK5                ------------------------------------------------------------
ATK1                ------------------------------------------------------------
Os03g64415          ------------------------------------------------------------
Os04g53760          ------------------------------------------------------------
Os07g01490          ------------------------------------------------------------
At2g22610           -NQSEKQYAQLQE------RLKSRDEICSNLQQKVKELECKLRERHQSDSAA--------
Os03g02290          -HSQITSEKQQNELFGKLKEKEEMCTTLQQKIAE--------------------------
At1g72250           -KLARQHVDTKIAEQQTKQQTEDENNTSKRPPLTNILLGSASK-----------------
Os12g42160          -KIARQHVDNKIAQ----------------------------------------------
At5g27550           -KTRIKQESRALATASSTTTTTSRHLRETLPTIIEK------------------------
Os05g33030          -KAARDTARST-------------------------------------------------


Os06g11380          SNKQTNAQGRDIGRSDSTKSQSSDVFPLPVSQDKAGNSGAIVKSSNELTKTTPAGEYLTS
AtKCA1              SLRASPNI-QPANVNSRGEGYSAEAVALPSTPNKNNGAITLVKSGTDLVKTTPAGEYLTA
AtKCA2              SSKASPTV-QPADVDSAG--------TLPSSVDKNEGTITLVKSSSELVKTTPAGEYLTA
```

```
AtKinesin-12A    --------------PVGATPVLKSPTLSVSPTIRNSRKSLKTSELSTASQKDSEGENLVT
At3g10310        -------------------------------PLASRRAQILSLEGPMSCKNEENGKGDPT
Os01g14090       -----------------------------SVTKLNRDHATIHDSIDGFNHHIMHQGSVMQ
At1g63640        -----------------------------------GRGTSDVDNCSEYSSKHSDSGSQQS
At5g41310        -----------------------------IHRHQNESRSSSKFSGGAKDNNIFEDTELLG
Os02g13580       ---------------------------------VGSGAASDLDNFSDTSDRQSEAGSMLS
Os06g36080       ---------------------------------VGSRAGSDVDNFSDISDRHSEAGSMQS
Os01g54080       ----------------------------------------------------EAEDNASD
Os05g44560       -----------------------------------------------YAEVNRDGGPTSY
At1g18410        ------------------------------------------------RHSVTDGEALAS
At1g73860        --------------------------------------------SSQSRYSVTDGESLAS
Os11g44880       -----------------------------DTSSWLDTSSQKEAALGEWVNNSQFGSSNSL
OsKCH1           -----------------------------NSSDLAENCNGIQKTDRMAVGNNQFENGNSI
Os03g18980       -----------------------------KKPSFDLQDLLASNDSPSWPDSISRANFQMG
At2g47500        -----------------------------------------TVNSPPWPPVASPGQAYRE
At1g09170        -----------------------------QSDSASSLDLQGLVGSPSWKTPPRDGKEEDM
ATK4             -----------------------------ASSRRHSLDIHELMKSSSPAWPRQPLNGKDE
AtKP1            -----------------------------ASPRMPNLAEERLNPSPSRRSLSTDRASTIK
OsKCH2           -----------------------------SGKQRRFRSPLSVRELDEKSPVINRELYLSA
AtKCBP           ------------------------------------------------------------
Os04g57140       ------------------------------------------------------------
At1g55550        -----------------------------SSSFKSRRNSMISVRAESACLPVKKKKNRFD
Os01g15540       -----------------------------WQSECSSECSMSLTSDMNWTPSIRDGTECSQ
At5g27950        ----------------------------------SSDKLVKIRKSSGFVPRFMTSTVASR
Os11g42800       -----------------------------QATEKTKSRASHGSVPHFMSPTVCSRQRHSS
ATK2             ------------------------------------------------------------
ATK3             ------------------------------------------------------------
ATK5             ------------------------------------------------------------
ATK1             ------------------------------------------------------------
Os03g64415       ------------------------------------------------------------
Os04g53760       ------------------------------------------------------------
Os07g01490       ------------------------------------------------------------
At2g22610        --------------NNQ------------KVKDLENNLKESEGSSLVWQQKVKDYENKLK
Os03g02290       -----------------------------ESEHKLRLQQQSESEIKELELKLKEQEHHRS
At1g72250        -----------------------------EMVNLTRPSLLESTTSYDLAPLPSGVPKYND
Os12g42160       -----------------------------DHLHQQQQSKKPENSPCPTRSPMAERNLNST
At5g27550        -----------------------------KPPLAPTRMRMPLRRITNFMPQQQPSQGHSK
Os05g33030       -----------------------------KPPLAPMRQRPPLGRIGNHIPPKAPLRLRLS


Os06g11380       ALMDFDPNQFEGVAAIADGANKLLMLPYFHCHRDYNETPPISDWCMVLAAVIKAGAAREH
AtKCA1           ALNDFDPEEYEGLAAIADGANKLLML--------------------VLAAVIKAGASREH
AtKCA2           ALNDFDPEQYEGLAAIADGANKLLML--------------------VLAAVIKAGASREH
AtKinesin-12A    EAADPSPATSKKMNNCSSALSTQKSKVFPVRTERLASSLHKGIKLLESYCQSTAQRRSTY
At3g10310        MEVHQLKNPRSPLSSYQNRA----------------------------------------
Os01g14090       MSATSSEDPVREETEKIITTVDTVPFCGLHP-----------------------------
At1g63640        SDERKHQKDYHQPSKFAGAAKGIDFDD---------------------------------
At5g41310        FEESNNEERLSDISDSCLSMG---------------------------------------
Os02g13580       VDPEISGLADVDSDGRLS------------------------------------------
Os06g36080       VDDIQQSREIMGLSKLSMSEM---------------------------------------
Os01g54080       DGCSVGETEYSPAGASET------------------------------------------
Os05g44560       TDITPTCLDEADFEDNASEDGFSG------------------------------------
At1g18410        STDAEYDDETEGSTDAPCAA----------------------------------------
At1g73860        SAEAEYDERLSEIT----------------------------------------------
Os11g44880       LELGPDATQDVVFYQRNSPEPQW-------------------------------------
OsKCH1           LELEPGATQLPTFFYQRYDPDKQR------------------------------------
Os03g18980       EERVTIGGEWIDKVVVNNNNSVG-------------------------------------
At2g47500        DDRSFGSSEWVDKVMVNNRQDEMR------------------------------------
At1g09170        EFIIPGSEWVDKHEDEITRSSKPENR----------------------------------
ATK4             DRESKSGEWIDKHEELIQNQNPNS------------------------------------
AtKP1            SRNKPDVTQNLPVSRTPFPARVPVV-----------------------------------
OsKCH2           KFKTPSPPVRSSLSAERVGIAKSV------------------------------------
AtKCBP           ------------------------------------------------------------
Os04g57140       ------------------------------------------------------------
At1g55550        SACDSSDRSVSKSTSIMRQNTADDAT----------------------------------
Os01g15540       DASEYEIKQVIFSEHEKSSHDQVTCYTDY-------------------------------
At5g27950        QRQTMSEKEINAKAQSIRS-----------------------------------------
Os11g42800       ASHSATKTRLTKSV----------------------------------------------
ATK2             ------------------------------------------------------------
ATK3             ------------------------------------------------------------
ATK5             ------------------------------------------------------------
```

```
ATK1                 ------------------------------------------------------------
Os03g64415           ------------------------------------------------------------
Os04g53760           ------------------------------------------------------------
Os07g01490           ------------------------------------------------------------
At2g22610            ESEGNSLVWQQKIKELEIKHKDE-------------------------------------
Os03g02290           VAESKIKELELKLKEQEHHRSVAESKAME-------------------------------
At1g72250            LSEKENNPEMADQVHLPNKTGRFS------------------------------------
Os12g42160           AEKPVTLLKDLGIARQMFSDSNTDTY----------------------------------
At5g27550            RFSDTTFKENNNSNRRSSSMDVNTLMK---------------------------------
Os05g33030           KAPTIQNKENIPVMLNKGSSGADTSKAVA-------------------------------


Os06g11380           EILAEIRDAVFSFIRKMEPRKVMDTMLVSRVKILYIRSLLARS----PELQSIKVSPVER
AtKCA1               EILAEIRDSVFSFIRKMEPRRVMDTMLVSRVRILYIRSLLARS----PELQTIRVSPVEC
AtKCA2               EILAEIRDSVFSFIRKMEPRRVMDTMLVSRVRILYIRSLLARS----PELQSIKVSPVER
AtKinesin-12A        RFSFKAPDSEPSTSISKADAGVQTIPGADAISEENTKEFLCCKCKCREQFDAQQMGDMPN
At3g10310            -----------------------------------------------VKVDGRTSIPQLQ
Os01g14090           -----------------------------------------------DAYISSKQSGLDT
At1g63640            -----------------------------------------------EDVELVGLADADS
At5g41310            -----------------------------------------------TETDGSISSGAME
Os02g13580           -----------------------------------------------DASDGISMGAEAD
Os06g36080           -----------------------------------------------GHNSVDPELPCFG
Os01g54080           ------------------------------------------------------------
Os05g44560           -----------------------------------------------GTDYSVGCAAGAS
At1g18410            -----------------------------------------------EGRKPLKISDKPK
At1g73860            -----------------------------------------------SDAASMGTQGSID
Os11g44880           -----------------------------------------------SWAGSVATEDSDD
OsKCH1               -----------------------------------------------RRAEPVETDDSDS
Os03g18980           -----------------------------------------------DWEGDSAALPDFF
At2g47500            -----------------------------------------------RVESLWGGATTEN
At1g09170            -----------------------------------------------AHTQLEKRTSSLK
ATK4                 -----------------------------------------------PEQFYQSMVPQQQ
AtKP1                -----------------------------------------------KSFSTVPLNPSAE
OsKCH2               -----------------------------------------------ERSENIDCTPVSR
AtKCBP               ------------------------------------------------------------
Os04g57140           ------------------------------------------------------------
At1g55550            ---------------------------------VYSQDISECD----IKLVVSEHKPKPL
Os01g15540           -----------------------------------------------PLAESRDIQIKIE
At5g27950            -----------------------------------------------VAKNLTQFSTSQS
Os11g42800           -----------------------------------------------NRYPAAELSGSHS
ATK2                 ------------------------------------------------------------
ATK3                 ------------------------------------------------------------
ATK5                 ------------------------------------------------------------
ATK1                 ------------------------------------------------------------
Os03g64415           ------------------------------------------------------------
Os04g53760           ------------------------------------------------------------
Os07g01490           ------------------------------------------------------------
At2g22610            -----------------------------------------------QSQEAVLLRQKIK
Os03g02290           -----------------------------------------------IGQELLETQRTEA
At1g72250            -----------------------------------------------ICAKRIPSAPAPR
Os12g42160           -----------------------------------------------SINHLMSMSSEKE
At5g27550            -----------------------------------------------PRRSSIAFRPAPA
Os05g33030           -----------------------------------------------GKARRVSLTPVIR


Os06g11380           FLEKSHTSRSRSSSRGSSPGRSPVHHHHDHGSRTSLIDEHVHGFKVNIKPERKSKFSSIV
AtKCA1               FLEKPNTGRSKSTSRGSSPGRSPVRY----------LDTQIHGFKVNIKAERRNKLASVV
AtKCA2               FLEKPYTGRTRSSSGSSSPGRSPVRY----------YDEQIYGFKVNLKPEKKSKLVSVV
AtKinesin-12A        LQLVPVDNSEVAEKSKNQVPKAVEKVLAGSIRREMALEEFCTKQASEITQLNRLVQQYKH
At3g10310            LLQTPVKGASRNDIQMISVDS---------------------------------------
Os01g14090           LLRTPCRSRNLNLEVGQTDEPSSSAK---------------------------LEKMTSS
At1g63640            EDRLSDISDSCLSMGTETDGSISSAV--------------------------------EL
At5g41310            LTLFPETSNPPEMFEQSEQN----------------------------------------
Os02g13580           SSVSNVADQEQEKTSNTA------------------------------------------
Os06g36080           YDDSEGRLSDISDSGLSMGAETDCSM------------------------------SSVV
Os01g54080           ------------------------------------------------------------
Os05g44560           VFPNSCSDRT--------------------------------------------------
At1g18410            PVTPR-------------------------------------------------------
At1g73860            VTKRPPRISDRAKSVTAK------------------------------------------
Os11g44880           FEVTTSCSSEQDMVRPTSAPKAPGSAN---------------------------------
OsKCH1               FDAATSSPSDQEMLLSTSGLKADGIA----------------------------------
```

```
Os03g18980          YQRYHSGTRDKQYLRNNSRKKDGNEF---------------------------EQQRPRF
At2g47500           GIGILPEDFYRRDLASDTSRIFSEHS-------------------------------YNI
At1g09170           REATRGVDKNKCNSSVDKGLEVRKIP----------------------------------
ATK4                SLYGGKQDFEVQSITDNESDE---------------------------------------
AtKP1               NNHRLHTDNSSEAFQNHQKLSARKLF--------PEIEEEHIRHALHIRQGGVKKTRAES
OsKCH2              IEVPPKVQHSSSRKTPSSVLTAQSLR-----------------KFRDSEENRSAKPSVRE
AtKCBP              ------------------------------------------------------------
Os04g57140          ------------------------------------------------------------
At1g55550           QMGPGSATKSRSNISNFEKDVMQKIGGTEFSRINSWLRSQSENRSYVLDKTQLPATHFLE
Os01g15540          EKGIVDIDNWLHQQIVEKTSTFRSKM----------------------VLDIPGVTEAEI
At5g27950           LSLSDSRSKALLRRSYTKPLQAA-------------------------------------
Os11g42800          FSYSSCKNAAKA------------------------------------------------
ATK2                ------------------------------------------------------------
ATK3                ------------------------------------------------------------
ATK5                ------------------------------------------------------------
ATK1                ------------------------------------------------------------
Os03g64415          ------------------------------------------------------------
Os04g53760          ------------------------------------------------------------
Os07g01490          ------------------------------------------------------------
At2g22610           ELEMRLKEQEKHIQEMATTREFPEVA--------------------NATPNEVKTCFKED
Os03g02290          MLQIKPRDLENNLQERTTLQDTNMILDSTNCMRVASTPGEAKAHLLTREEAMSEKEQHIL
At1g72250           RSSLAPTTSTSREMVYLTRPPLSEST-----------------TSYDLPPLPNGGLKYSD
Os12g42160          NNPAGGAQPTKARRVSLCGGAHQQPA----------------------APPRRGSLIPLP
At5g27550           PSAIASSNKTIMPRRRVSIATLRPEP-----------------------------SSLSS
Os05g33030          HIPLQPKRRSSLAVLPTQREQLSIFP-----------------------------DKRSV


Os06g11380          LKLRGIEEETWRQHVTGGKLREITEEAKAFAIGNKALAALFVHTPAGELQRQIRAWLAEN
AtKCA1              SRMRGLEQDAGRQQVTGVKLREMQDEAKSFAIGNKALAALFVHTPAGELQRQIRLWLAEN
AtKCA2              SRIRGHDQDTGRQQVTGGKLREIQDEAKSFAIGNKPLAALFVHTPAGELQRQIRSWLAES
AtKinesin-12A       ERECNAIIGQTREDKIIRLESLMDGVLSKEDFLDEEFASLLHEHKLLKDMYQNHPEVLKT
At3g10310           -----------KTNGKGSHIRKSLRTIGKLINGSEK------------------------
Os01g14090          NATKKGSHLRKSIQSSIGKLIHGSERRNVQHLGQATPAKIANSTN---------------
At1g63640           TLFPETAKPLELIERPEARMTSEKLEKSVKMGKTEPK-----------------------
At5g41310           ------------------------------------------------------------
Os02g13580          ------------------------------------------------------------
Os06g36080          ELTSLPDQDRVSGTQKEQHMAPSTPKDRLHKVATRA------------------------
Os01g54080          ------------------------------------------------------------
Os05g44560          ------------------------------------------------------------
At1g18410           ------------------------------------------------------------
At1g73860           ------------------------------------------------------------
Os11g44880          ------------------------------------------------------------
OsKCH1              ------------------------------------------------------------
Os03g18980          YSTNTDDSDDIDIATSDSSESDALWQFNVQSINSSIS-----------------------
At2g47500           FMGNNNSTDDLDAATSDSSEPDLLWQFNQSTKIPTRSN----------------------
At1g09170           --YEEEANESDETATSDCSETNLMWQLNVQVNMPRPASNG--------------------
ATK4                ------------AATSDCSDSDLLWRLSVQVNVPK-------------------------
AtKP1               SKAKAKQPSPARFQKLDVGISLRSDADSEAKVGNYQTQKGNNNHNVIHSRFQNFDVGISL
OsKCH2              SMTKTRLDSATKPPQKEEQTANKNTGTRVRSEAKIPRNISDIENEFANSEPTFH------
AtKCBP              ------------------------------------------------------------
Os04g57140          ------------------------------------------------------------
At1g55550           NLNRSLEKSPTQSFTTEKITGNELEGIEETKTNETVVNPTLMLKKLFELQCLCSAE----
Os01g15540          HVSSIPSPTTMACTKEDSQVKDEVMGLTLQSSTDYVEDI---------------------
At5g27950           ------------------------------------------------------------
Os11g42800          ------------------------------------------------------------
ATK2                ------------------------------------------------------------
ATK3                ------------------------------------------------------------
ATK5                ------------------------------------------------------------
ATK1                ------------------------------------------------------------
Os03g64415          ------------------------------------------------------------
Os04g53760          ------------------------------------------------------------
Os07g01490          ------------------------------------------------------------
At2g22610           NFGNENMESNTNILRTSNRLKTKRHDSLNLNEMTRKKRASRSGETE--------------
Os03g02290          RSSDSMNKKVTNNSSIVGAPEVVNEKKRKGDARNSSIGGELENQPV--------------
At1g72250           LIEKVNNQEMAEQVQIPKRIGAGRSSICAKRIPPAPRRKSFAPMPFIPITSTLTSPDEKS
Os12g42160          RRNSLMLPLPLPKPATPAAAASPLDMITEQCSSPLVIAPNDIRGGGG-------------
At5g27550           METPSRPPPSFRGDPRKARYSKLFSPDRNLVTPNAMKSSRFMKSPL--------------
Os05g33030          SRLSHIQMPRRSIATFNSIPATPLAAAAHKQVDGTPEARQLRRIE---------------


Os06g11380          FEFLSVTGGDVAG-ASGQLELLSTAIMDGWMAGLGTARPPSTDALGQLLSEYTKRVYTSQ
```

```
AtKCA1          FEFLSVTSDDVSGGNGGQLELLSTAIMDGWMAGLGAAVPPHTDALGQLLSEYAKRVYTSQ
AtKCA2          FEFLSVTADDVSGVTTGQLELLSTAIMDGWMAGVGAAVPPHTDALGQLLSEYAKRVYTSQ
AtKinesin-12A   KIELERTQEEVENFKNFYGDMGEREVLLEEIQDLKLQLQCYIDPSLKSALKTCTLLKLSY
At3g10310       ------------------------------------------------------------
Os01g14090      ------------------------------------------------------------
At1g63640       ------------------------------------------------------------
At5g41310       ------------------------------------------------------------
Os02g13580      ------------------------------------------------------------
Os06g36080      ------------------------------------------------------------
Os01g54080      ------------------------------------------------------------
Os05g44560      ------------------------------------------------------------
At1g18410       ------------------------------------------------------------
At1g73860       ------------------------------------------------------------
Os11g44880      ------------------------------------------------------------
OsKCH1          ------------------------------------------------------------
Os03g18980      ------------------------------------------------------------
At2g47500       ------------------------------------------------------------
At1g09170       ------------------------------------------------------------
ATK4            ------------------------------------------------------------
AtKP1           FSDLCAGDKSDSTLKS--------------------------------------------
OsKCH2          ------------------------------------------------------------
AtKCBP          ------------------------------------------------------------
Os04g57140      ------------------------------------------------------------
At1g55550       ------------------------------------------------------------
Os01g15540      ------------------------------------------------------------
At5g27950       ------------------------------------------------------------
Os11g42800      ------------------------------------------------------------
ATK2            ------------------------------------------------------------
ATK3            ------------------------------------------------------------
ATK5            ------------------------------------------------------------
ATK1            ------------------------------------------------------------
Os03g64415      ------------------------------------------------------------
Os04g53760      ------------------------------------------------------------
Os07g01490      ------------------------------------------------------------
At2g22610       ------------------------------------------------------------
Os03g02290      ------------------------------------------------------------
At1g72250       ------------------------------------------------------------
Os12g42160      ------------------------------------------------------------
At5g27550       ------------------------------------------------------------
Os05g33030      ------------------------------------------------------------

Os06g11380      LHHLKDIAGTLATEVADDPAHVSKLRSALESVDHKRRKIMQQMRTDTVLLTKEEGGSPIR
AtKCA1          MQHMKDIAGTLAAEEAEDAGQVSKLRSALESVDHKRRKILQQMKSDAALLNLEEGSSPIP
AtKCA2          MQHLKDIAGTLASEEAEDAGQVAKLRSALESVDHKRRKILQQMRSDAALFTLEEGSSPVQ
AtKinesin-12A   QAPPVNAIPESQDESLEKTLEQERLCWTEAETKWISLSEELRTELEASKALINKQKHELE
At3g10310       ----------------------------------------------------RKENIPAD
Os01g14090      ----------------------------------------------------NDVPSSIT
At1g63640       ----------------------------------------------------DRTNIPSK
At5g41310       ----------------------------------------------------DKAHVGVG
Os02g13580      ----------------------------------------------------AKERLTRA
Os06g36080      ----------------------------------------------------SRTTTPKT
Os01g54080      ----------------------------------------------------SAERAHKA
Os05g44560      ----------------------------------------------------ADTSIRRI
At1g18410       ----------------------------------------------------SNTTTSRP
At1g73860       ----------------------------------------------------SSTSVTRP
Os11g44880      ----------------------------------------------------GSASIARK
OsKCH1          ----------------------------------------------------SRGAFIIK
Os03g18980      ----------------------------------------------------ENGSKIKK
At2g47500       ----------------------------------------------------IESKLKKP
At1g09170       ----------------------------------------------------SSTKLKKN
ATK4            ----------------------------------------------------VSNIQNSA
AtKP1           ----------------------------------------------------DSSETDNE
OsKCH2          ----------------------------------------------------SNRKAWKL
AtKCBP          ------------------------------------------------------------
Os04g57140      ------------------------------------------------------------
At1g55550       ----------------------------------------------------EEDQILSR
Os01g15540      ----------------------------------------------------KQSKTDNQ
At5g27950       ----------------------------------------------------ANSGTPPE
Os11g42800      ----------------------------------------------------RSVAFSSS
ATK2            ------------------------------------------------------------
```

```
ATK3                ------------------------------------------------------------
ATK5                ------------------------------------------------------------
ATK1                ------------------------------------------------------------
Os03g64415          ------------------------------------------------------------
Os04g53760          ------------------------------------------------------------
Os07g01490          ------------------------------------------------------------
At2g22610           ----------------------------------------------------NNGDDPQM
Os03g02290          ----------------------------------------------------GSQNASRK
At1g72250           ----------------------------------------------------GANQVLCT
Os12g42160          ----------------------------------------------------GGGRNKRI
At5g27550           ----------------------------------------------------GGGGSSWK
Os05g33030          ----------------------------------------------------FSSSKFRS

Os06g11380          NPPTAAEDARLASLISLDNIIKQVKEVMRQSSARPLRKSKKKALLESLDDLLAQMPSLLD
AtKCA1              NPSTAAEDSRLASLISLDGILKQVKEITRQASVHVLSKSKKKALLESLDELTERMPSLLD
AtKCA2              NPSTAAEDSRLASLISLDAILKQVKEITRQASVHVLSKSKKKALLESLDELNERMPSLLD
AtKinesin-12A       IEKRCGEELKEAMQMAMEGHARMLEQYADLEEKHMQLLARHRRIQDGIDDVKKAAARAGV
At3g10310           PRSPLGVANNFSHIKSPDTSN---------------------------------------
Os01g14090          PDLRLRRRQSLTGLPPPPSTMS--------------------------------------
At1g63640           IPKQTLKPPGQTRP----------------------------------------------
At5g41310           PSKPLKHTPKPDISK---------------------------------------------
Os02g13580          VNRVQKLTLPKAGQSSSL------------------------------------------
Os06g36080          PQSPTLWPK---------------------------------------------------
Os01g54080          PSRITRFFLTKNGQPSTS------------------------------------------
Os05g44560          SSRIARFSLTKNGQPA--------------------------------------------
At1g18410           LDKLKQVTMRTT------------------------------------------------
At1g73860           LDKLRKVATRTTS-----------------------------------------------
Os11g44880          AQPKGAKSTDIRSTNPAKRAAP--------------------------------------
OsKCH1              KPQTKNTKITATKIPNLA------------------------------------------
Os03g18980          PQTKLRESSDTRTPLH--------------------------------------------
At2g47500           VSKPIRSPQSRNNSNNT-------------------------------------------
At1g09170           QSKISRVAAETRSMIPSL------------------------------------------
ATK4                NPKPKKIQPRTAKL----------------------------------------------
AtKP1               PPSKSKNAQRNSSKNSLNHKLRTI------------------------------------
OsKCH2              PPQSTRQSQSIDLRASVREM----------------------------------------
AtKCBP              ------------------------------------------------------------
Os04g57140          ------------------------------------------------------------
At1g55550           FPIPGYEDDDESRYPPILENDGFSQHIDNEWFGVNNYSADWERDSPATIPLLECEPDLKQ
Os01g15540          FTAKELCTPPFKEFSSNNEVKGHKNEHPVY------------------------------
At5g27950           TPKRHIKD----------------------------------------------------
Os11g42800          MPKMKCLPLKSDQIN---------------------------------------------
ATK2                ------------------------------------------------------------
ATK3                ------------------------------------------------------------
ATK5                ------------------------------------------------------------
ATK1                ------------------------------------------------------------
Os03g64415          ------------------------------------------------------------
Os04g53760          ------------------------------------------------------------
Os07g01490          ------------------------------------------------------------
At2g22610           KEKRIRKSDPPKVFSRVV------------------------------------------
Os03g02290          RSLQGEPRLKRKSTEPLKN-----------------------------------------
At1g72250           SPKLHRSNGKTLTSILRRSIQKRMQMKP--------------------------------
Os12g42160          INSILRRSLQKKVIIRPPLMAAHQSGRRAGAGV---------------------------
At5g27550           PSHPTVIALQKKAV----------------------------------------------
Os05g33030          PPALARFNSRNNALSPQQKLRLASGSGNASKIC---------------------------

Os06g11380          VDHPCAQKQIMEARKVVESLQEDPDE---------PATDLNSNTLGESEVSQWNVLQFNT
AtKCA1              IDHPCAQREIATAHQLVETIPEQEDTNILEQSHDRRPSLESISSGETDVSQWNVLQFNT-
AtKCA2              VDHPCAQREIDTAHQLVETIPEQED----NLQDEKRPSIDSISSTETDVSQWNVLQFNTG
AtKinesin-12A       RGAESRFINALAAEISALKVEKEKERQYLRDENKSLQTQLRDTAEAIQAAGELLVRLKEA
At3g10310           -----------------------------------AKTMRRQSLTGVMPPGQERSRRSSI
Os01g14090          -----------------------------------RRSSLGGKSDIGAKNSPISACAASE
At1g63640           -----------------------------------SRLSIATSSSSKALTGAKRPTISTS
At5g41310           -----------------------------------PSRLSISTTSSKALTSSKRPVTGIS
Os02g13580          -----------------------------------RPKPRDPAPARSSAATGVRKSSTSQ
Os06g36080          -----------------------------------LRDPPPPRSPISTSTGKVRVTQATS
Os01g54080          -----------------------------------RPKPREVVPKTQGSMRPGTAQATGG
Os05g44560          -----------------------------------TSRPKPKDTAPKTPNQTRVQSSQLI
At1g18410           -----------------------------------NIAKATSALLSPSSQGMKKTGSASN
At1g73860           -----------------------------------TVAKVTGLTSSSKGLASSSIKKTGS
```

```
Os11g44880          -----------------------------------LQKKINGPPSASTKNGKQLSLSAAD
OsKCH1              -----------------------------------MKSPMSEKRLQTPIRNSKQLPFSTT
Os03g18980          -----------------------------------SQIPSASRKTSNGNRSGRQPLSGSD
At2g47500           -----------------------------------VSRPLASQKVGNGPRGMKQFGPADM
At1g09170           -----------------------------------IPTPTRTLSLGAAISSPGQTSSRHN
ATK4                -----------------------------------SETRSLIPSLIPAPSKRPPNTVNSQ
AtKP1               -----------------------------------YAHEDTSLVDDKPSNGTAHIKEGNN
OsKCH2              -----------------------------------EPLTEGKPRRSKAPHGDRTNVPLPE
AtKCBP              ------------------------------------------------------------
Os04g57140          ------------------------------------------------------------
At1g55550           LLPELGLDRSL------------------------KPRGLAVAEGAAPPLLRAQETLGER
Os01g15540          -----------------------------------HGRPRRSLQEELENCTLEKPNMDSK
At5g27950           -----------------------------------NSLQRKNMNDTSSPRSKMVTSSDPN
Os11g42800          -----------------------------------MSNNSIDSTAASAPRRRESFISRPA
ATK2                ------------------------------------------------------------
ATK3                ------------------------------------------------------------
ATK5                ------------------------------------------------------------
ATK1                ------------------------------------------------------------
Os03g64415          ------------------------------------------------------------
Os04g53760          ------------------------------------------------------------
Os07g01490          ------------------------------------------------------------
At2g22610           -----------------------------------RPTRTASGSSSQVPVAQKRVIKREQ
Os03g02290          -----------------------------------PGRVTATSKTAAATHKTGPVTRATR
At1g72250           -----------------------------------SPRQQPMRRGGGINVGMERVRLSIG
Os12g42160          -----------------------------------AGTTTHGGGGGGVMRARRVPVSGGR
At5g27550           -----------------------------------VWSPLKFKNRRPSLVAIRSSASSSS
Os05g33030          -----------------------------------FSVQKRVILGSPAPVKSSLLSGTGI


Os06g11380          GTSAPFIIKCGANSSCELVIKADQKIQEPKGDEIIRVVPKPSVLAEMSFEEIKGVFEELP
AtKCA1              GSSAPFIIKCGGNNNSELVIKADARVQEPKGGEIVRVVPRPSVLVNMSLEEMKQMFVQLP
AtKCA2              GSSAPFIIKCGANSNSELVIKADARIQEPKGGEIVRVVPRPSVLENMSLEEMKQVFGQLP
AtKinesin-12A       EEGLTVAQKRAMDAEYEAAEAYRQIDKLKKKHENEINTLNQLVPQSHIHNECSTKCDQAV
At3g10310           GGKPIENGKKDHVFTPFRLNKFNN------------------------------------
Os01g14090          YKPLPFSWVRWPEQYQCSDKRGAKTPPPVNSAAKAKRWL---------------------
At1g63640           SSAKPLNRRR--------------------------------------------------
At5g41310           SSVKPLNRKR--------------------------------------------------
Os02g13580          ATPLARNNSTLKRGP---------------------------------------------
Os06g36080          SSRNSSTQKRWT------------------------------------------------
Os01g54080          SLAKPSKRR---------------------------------------------------
Os05g44560          GGSSLRASKRWQK-----------------------------------------------
At1g18410           FLKSPKDSKRWS------------------------------------------------
At1g73860           TSSLAKSSKRWA------------------------------------------------
Os11g44880          GKRAPNGKVSAKK-----------------------------------------------
OsKCH1              GGRRTRNGKINTPK----------------------------------------------
Os03g18980          SRRLSSNGRHAGTK----------------------------------------------
At2g47500           KRKATNARH---------------------------------------------------
At1g09170           NSTVVVKKRQNPK-----------------------------------------------
ATK4                PQRPTRDGKRRLSLGT--------------------------------------------
AtKP1               NISMPEFRRSRSTHHARFMVP---------------------------------------
OsKCH2              TRRSVSLPRGKMALV---------------------------------------------
AtKCBP              ------------------------------------------------------------
Os04g57140          ------------------------------------------------------------
At1g55550           GKGPTFMQKLQALCFRILLGLGFMDVGFGNDFFNGLTK----------------------
Os01g15540          SHRSHDDKHKTGKFTKFFQALQTAWIGALLALGTVSIGLEHGFFQSLTL-----------
At5g27950           VRAKLCHHKRRMSSLT--------------------------------------------
Os11g42800          QRAPLHQHRRRMSSLT--------------------------------------------
ATK2                ------------------------------------------------------------
ATK3                ------------------------------------------------------------
ATK5                ------------------------------------------------------------
ATK1                ------------------------------------------------------------
Os03g64415          ------------------------------------------------------------
Os04g53760          ------------------------------------------------------------
Os07g01490          ------------------------------------------------------------
At2g22610           QEVPVVKERDSKKKIWSR------------------------------------------
Os03g02290          QQPAVNKTRGWVR-----------------------------------------------
At1g72250           NRGRLAHRVLLTNARKAGLKETPQKQERWI------------------------------
Os12g42160          GGGGVQHNREKERGWNNGTSLRQLN-----------------------------------
At5g27550           ASDLLRREQ---------------------------------------------------
Os05g33030          FNPALREKMMAAKIGNAQRVFNTNRRKSVL------------------------------
```

```
Os06g11380         EAISLLALARTADGTRARYSRLYRTLANKVPALKDIVAEMEKGGVFKDVRS-
AtKCA1             EALSLLALARTADGTRARYSRLYKTLAMKVPSLKDLVSELE-----------
AtKCA2             EALSSLALARTADGTRARYSRLYRTLAMKVPSLRDLVGELEKGGVLKDTKST
AtKinesin-12A      EPSVNASSEQQWRDEFEPLYKKETEFSNLAEPSWFSGYDRCNI---------
At3g10310          ----------------------------------------------------
Os01g14090         ----------------------------------------------------
At1g63640          ----------------------------------------------------
At5g41310          ----------------------------------------------------
Os02g13580         ----------------------------------------------------
Os06g36080         ----------------------------------------------------
Os01g54080         ----------------------------------------------------
Os05g44560         ----------------------------------------------------
At1g18410          ----------------------------------------------------
At1g73860          ----------------------------------------------------
Os11g44880         ----------------------------------------------------
OsKCH1             ----------------------------------------------------
Os03g18980         ----------------------------------------------------
At2g47500          ----------------------------------------------------
At1g09170          ----------------------------------------------------
ATK4               ----------------------------------------------------
AtKP1              ----------------------------------------------------
OsKCH2             ----------------------------------------------------
AtKCBP             ----------------------------------------------------
Os04g57140         ----------------------------------------------------
At1g55550          ----------------------------------------------------
Os01g15540         ----------------------------------------------------
At5g27950          ----------------------------------------------------
Os11g42800         ----------------------------------------------------
ATK2               ----------------------------------------------------
ATK3               ----------------------------------------------------
ATK5               ----------------------------------------------------
ATK1               ----------------------------------------------------
Os03g64415         ----------------------------------------------------
Os04g53760         ----------------------------------------------------
Os07g01490         ----------------------------------------------------
At2g22610          ----------------------------------------------------
Os03g02290         ----------------------------------------------------
At1g72250          ----------------------------------------------------
Os12g42160         ----------------------------------------------------
At5g27550          ----------------------------------------------------
Os05g33030         ----------------------------------------------------
```
